# The effect of local non‐thermal plasma therapy on the cancer‐immunity cycle in a melanoma mouse model

**DOI:** 10.1002/btm2.10314

**Published:** 2022-04-21

**Authors:** Abraham Lin, Joey De Backer, Delphine Quatannens, Bart Cuypers, Hanne Verswyvel, Edgar Cardenas De La Hoz, Bart Ribbens, Vasiliki Siozopoulou, Jonas Van Audenaerde, Elly Marcq, Filip Lardon, Kris Laukens, Steve Vanlanduit, Evelien Smits, Annemie Bogaerts

**Affiliations:** ^1^ PLASMANT‐Research Group University of Antwerp Antwerpen‐Wilrijk Belgium; ^2^ Center for Oncological Research (CORE), Integrated Personalized & Precision Oncology Network (IPPON) University of Antwerp Antwerpen‐Wilrijk Belgium; ^3^ Department of Biomedical Sciences University of Antwerp Antwerpen‐Wilrijk Belgium; ^4^ Adrem Data Lab, Department of Computer Science University of Antwerp Antwerpen Belgium; ^5^ Industrial Vision Lab (InViLab) University of Antwerp Antwerpen Belgium; ^6^ Department of Pathology University Hospital of Antwerp Antwerpen‐Wilrijk Belgium

**Keywords:** cancer‐immunity cycle, cancer therapy, cold atmospheric plasma, melanoma, non‐thermal plasma, plasma oncology

## Abstract

Melanoma remains a deadly cancer despite significant advances in immune checkpoint blockade and targeted therapies. The incidence of melanoma is also growing worldwide, which highlights the need for novel treatment options and strategic combination of therapies. Here, we investigate non‐thermal plasma (NTP), an ionized gas, as a promising, therapeutic option. In a melanoma mouse model, direct treatment of tumors with NTP results in reduced tumor burden and prolonged survival. Physical characterization of NTP treatment in situ reveals the deposited NTP energy and temperature associated with therapy response, and whole transcriptome analysis of the tumor identified several modulated pathways. NTP treatment also enhances the cancer‐immunity cycle, as immune cells in both the tumor and tumor‐draining lymph nodes appear more stimulated to perform their anti‐cancer functions. Thus, our data suggest that local NTP therapy stimulates systemic, anti‐cancer immunity. We discuss, in detail, how these fundamental insights will help direct the translation of NTP technology into the clinic and inform rational combination strategies to address the challenges in melanoma therapy.

## 1 INTRODUCTION

Skin cancer is the most common cancer worldwide, and melanoma is responsible for 65% of skin cancer‐related deaths.^1^ The incidence of melanoma is growing globally at a rate faster than other malignancies, and distressingly, the patient demographic includes younger individuals compared to other cancer types.^1^, ^2^ Early‐stage melanomas are usually treated with surgical excision, and in the majority of cases, treatment is curative.^3^ However, certain patients experience relapse and further dissemination of the disease. For more advanced stages, surgical resection is no longer an option and systemic treatment is required for managing metastasis.^4^, ^5^ While significant advancements in metastatic melanoma have been made in the last decade with the introduction of immune checkpoint blockades and targeted therapies, challenges still remain, including significant associated adverse effects, limited efficacious populations, and development of resistance.^6^ Therefore, novel therapeutic strategies and combinations are still required for both early‐ and late‐stage melanoma treatment.

Non‐thermal plasma (NTP) technology has been emerging onto the oncology scene, and medical NTP devices operate by ionizing a gas (e.g., argon, helium, air) at atmospheric pressure and room temperature. NTP systems for biomedical applications have been thoroughly characterized for in vitro systems, which include component analysis (e.g., pulsed‐electric fields, UV radiation),^7^, ^8^ gas‐phase measurements of excited and reactive species,^9^ and liquid chemistry quantification.^10^, ^11^ Preclinical research has reported the ability of NTP treatment to induce immunogenic cell death (ICD) in several cancer types including melanoma and even indicate abscopal effects in vivo.^12^, ^13^, ^14^, ^15^ Treatment of cancerous cells in vitro increased tumor immunogenicity through the emission of multiple danger signals such as surface‐exposed calreticulin (CRT), secreted adenosine triphosphate (ATP), and released high mobility group box protein 1 (HMGB1).^15^, ^16^, ^17^, ^18^ Furthermore, co‐culturing NTP‐treated cancer cells with ex vivo immune cells, particularly immature dendritic cells (DCs), increased DC maturation, cytokine release, and phagocytosis.^15^, ^18^, ^19^ Using the gold standard vaccination assay to test ICD‐inducing agents, our laboratory has also demonstrated that the short‐lived reactive oxygen species (ROS) generated by NTP, particularly •OH, •NO, O/O_3_, are the main effectors of NTP‐induced ICD.^13^ Thus, NTP is similar to other localized ROS therapies, such as photodynamic therapy^20^, ^21^ and electrochemical therapy,^22^, ^23^ in which harmful side effects can be modulated, while local anti‐cancer responses and systemic, immune responses can still be induced. Therefore, NTP is an attractive therapy for superficial skin cancers, which are easily accessible for treatment with the device.

While clinical pilot studies with NTP have only recently started, first results on cutaneous skin lesions indicate a promising treatment modality.^24^, ^25^, ^26^, ^27^ Metelmann et al. applied NTP to six patients with locally advanced squamous cell carcinoma of the oropharynx as part of their palliative treatment. Treatment not only reduced the need for pain medication and reduced odor in infected ulcers, but partial remission was observed in two patients for at least 9 months.^24^, ^25^, ^26^ Friedman et al. treated five patients diagnosed with actinic keratosis, which are pre‐cancerous skin lesions with the potential to develop into squamous cell carcinoma. After a single NTP treatment, 9 of the 17 treated lesions were fully resolved and 3 showed significant improvement in a 1‐month follow‐up.^27^ Most importantly, in both these clinical studies, no serious adverse effects were reported, which indicates the high tolerability of NTP therapy.

As promising clinical reports with NTP treatment are becoming available and more preclinical reports are focusing on the immunological implications of treatment, it is crucial and urgent to gain deeper insight into NTP treatment effects. Of note, a current gap in understanding is on how NTP therapy affects the cancer‐immunity cycle: a series of events directed by the patient's immune system, which leads to effective killing of cancerous cells.^28^ This understanding would provide application insight into how NTP can be used in the clinic and strategically combined with existing therapies to further enhance patient anti‐cancer immunity for greater clinical benefit.

In this study, we investigated the anti‐cancer effects of NTP treatment on the tumor and the cancer‐immunity cycle in an in vivo melanoma model. Physical characterization of NTP treatment was performed in situ and the delivered energy per NTP treatment pulse was measured to be approximately 0.9 mJ. Whole transcriptome sequencing and gene set enrichment analysis of the tumor following treatment revealed several up‐ and downregulated signaling, immune response, and stress pathways. The progression of anti‐cancer immunity was investigated by evaluating immune cell populations in the tumor, spleen, and tumor‐draining lymph nodes (TDLN) at discrete time points. Indeed, following treatment, cells of both the innate and adaptive immune system were stimulated toward a more anti‐cancer profile. Taken together, our results showed that local NTP application can elicit tumoricidal activity and stimulate systemic, anti‐cancer immune responses. This study provides crucial, fundamental insights into immunological activation following NTP treatment of melanoma tumors, which offers new perspective on its use in the clinic and on more rational combination strategies with existing therapies.

## 2 RESULTS

### 2.1 Physical characterization of the NTP device

A microsecond‐pulsed dielectric barrier discharge (DBD) plasma system was used for all experiments. While NTP treatment with a DBD plasma system has been performed in the past,^12^, ^29^, ^30^ direct comparisons with our system cannot be made due to the different electrical parameters of the systems used. Therefore, we first performed a pilot study on C57BL/6J mice bearing subcutaneous B16F10 melanoma tumors (*n* = 4–5) to determine the ideal, therapeutic NTP treatment intensity (Figure S1). From the study, the optimal NTP treatment parameters were determined (Table 1) and the thermal and electrical profile of this NTP regime was further investigated. Since NTP discharge characteristics are highly influenced by their treatment target,^31^ all measurements were performed in situ, on the surface of a mouse, analogous to treatment of tumors (details in Section 5).

**TABLE 1:** NTP system parameters for in vivo treatment

Microsecond‐pulsed DBD system	
Excitation	Microsecond pulse
Voltage	30 kV
Pulse frequency	700 Hz
Pulse rise time	1–1.5 μs
Pulse width	2 μs
Energy per pulse	0.9 mJ/pulse
Treatment time	10 s
Application distance	1–3 mm
Total energy of NTP treatment	6.3 J

**FIGURE 1:**
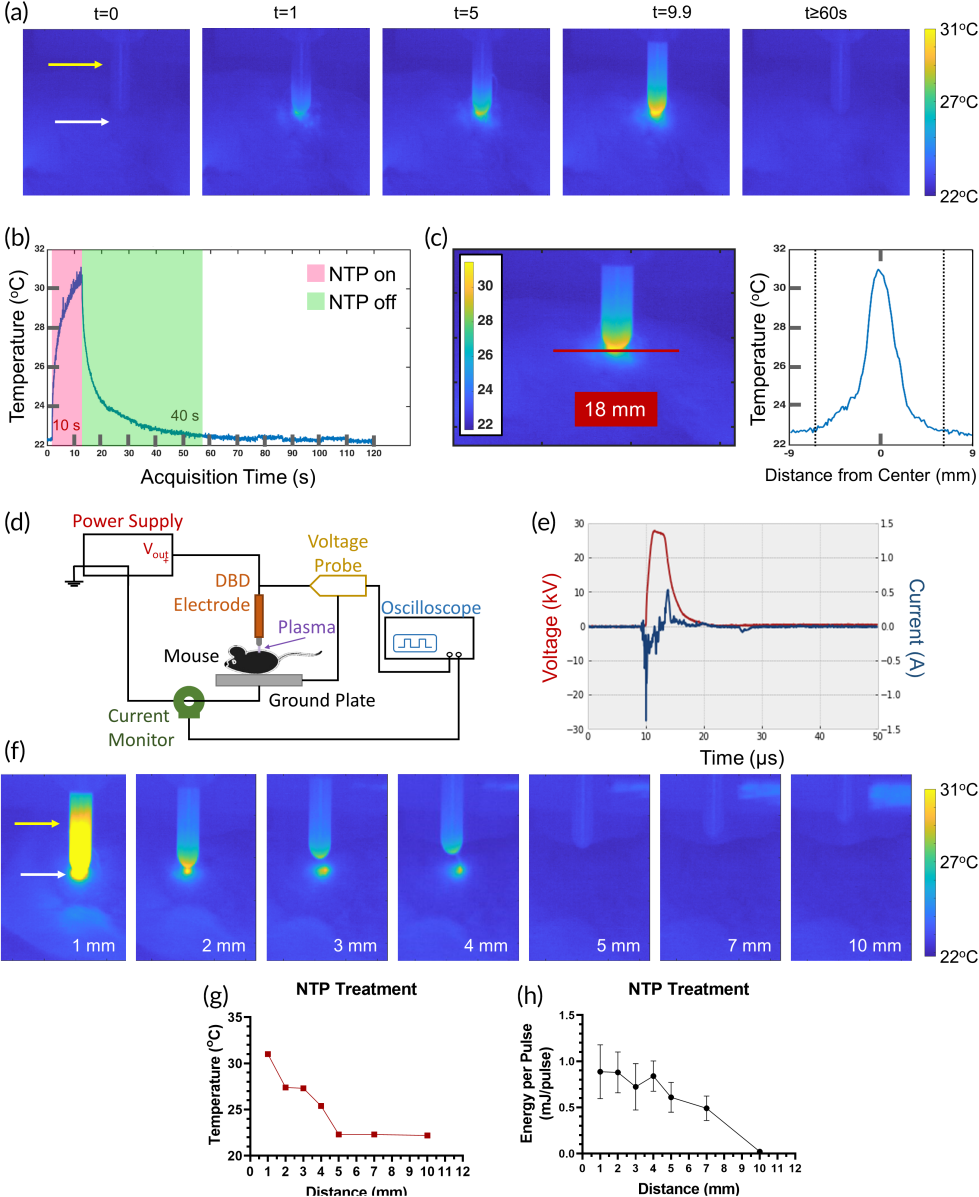
Temperature and energy characterization of non‐thermal plasma (NTP) treatment. (a) The development of the NTP discharge was observed, and the temperature of the treated mouse skin was monitored over time (t, seconds). (b) At the end of the 10 s treatment, the mouse skin reached 31°C but returned to baseline (22°C) within 40 s. (c) The spatial temperature profile was also measured across the skin under the NTP device (represented by the red line). The temperature was highest directly under the NTP device, while the skin 6.5 mm away (represented by the dotted lines) was unaffected. (d) The schematic setup used to measure the (e) voltage and current of a single NTP discharge on the surface of the mouse is shown. (f) The NTP applicator was adjusted over a range of distances and the (g) temperature and (h) energy per pulse was measured. Data are represented as mean ± SEM (*n* = 5–14). The yellow and white arrows in (a) and (f) indicate the position of the dielectric barrier discharge (DBD) electrode and skin surface, respectively

Using a high‐sensitivity, cooled thermal imaging camera, the temperature of the discharge and changes to the mouse skin were monitored over time, to determine whether treatment was thermally well‐tolerated. Selected thermal images are shown (Figure 1a) and the full image collection process is also provided (Video S1). At baseline, the temperature of the mouse skin was around 22°C, increased up to 31°C within 10 s of NTP treatment (NTP on), and returned to baseline within 40 s after treatment was completed (NTP off) (Figure 1b). Images of the skin taken immediately after NTP treatment also indicated no visible necrosis or thermal damage to the skin (Figure S2). Further tests showed that NTP treatment up to 1 min did not further increase the temperature of the skin (Figure S3 and Video S2). At approximately 6.5 mm away from the center of treatment, the temperature of the surrounding tissue was unaffected (Figure 1c). Altogether, the rise in temperature associated with NTP therapy is minimal. Therefore, these data suggest that NTP temperature is not associated with its therapeutic effect.

In our previous reports, we have indicated the importance of the delivered NTP treatment energy in dictating biological response in vitro.^32^ Therefore, we determined the NTP energy per pulse on the mouse during treatment (Figure 1d). This was calculated based on voltage and current measurements (Figure 1e) of a single pulse and after accounting for displacement current (details in Section 5). At a 1 mm application distance, the energy per pulse (*ε*_pulse_) on the mouse was 0.9 ± 0.3 mJ. Since the delivered energy of the plasma is highly subjected to the application distance, both the temperature and energy profiles were also studied for a range of distances. As the application distance increased, the temperature rise on the skin surface decreased, up to 5 mm where no changes were observed (Figure 1f,g). A similar trend was observed in the *ε*_pulse_ of NTP (Figure 1h). These data indicate that the stability of hand‐held NTP application, as well as the respiratory rate of the mouse, could contribute to the variation in treatment, though the effects appear minimal between 1 and 4 mm. To our knowledge, this is the first report to have studied the energy per pulse and temperature over a range of application distances in vivo to inform on the stability of local NTP application. Taken together, it is clear that the thermal properties of NTP in this regime are not responsible for its tumoricidal effects and that the therapy was thermally and electrically well‐tolerated. It is, in fact, likely that ROS generated by NTP therapy, in proportion to NTP treatment energy, are the main effectors of its anti‐cancer properties, which is in‐line with evidence from previous reports.^29^, ^30^, ^32^, ^33^

### 2.2 NTP therapy elicits anti‐tumor responses

**FIGURE 2:**
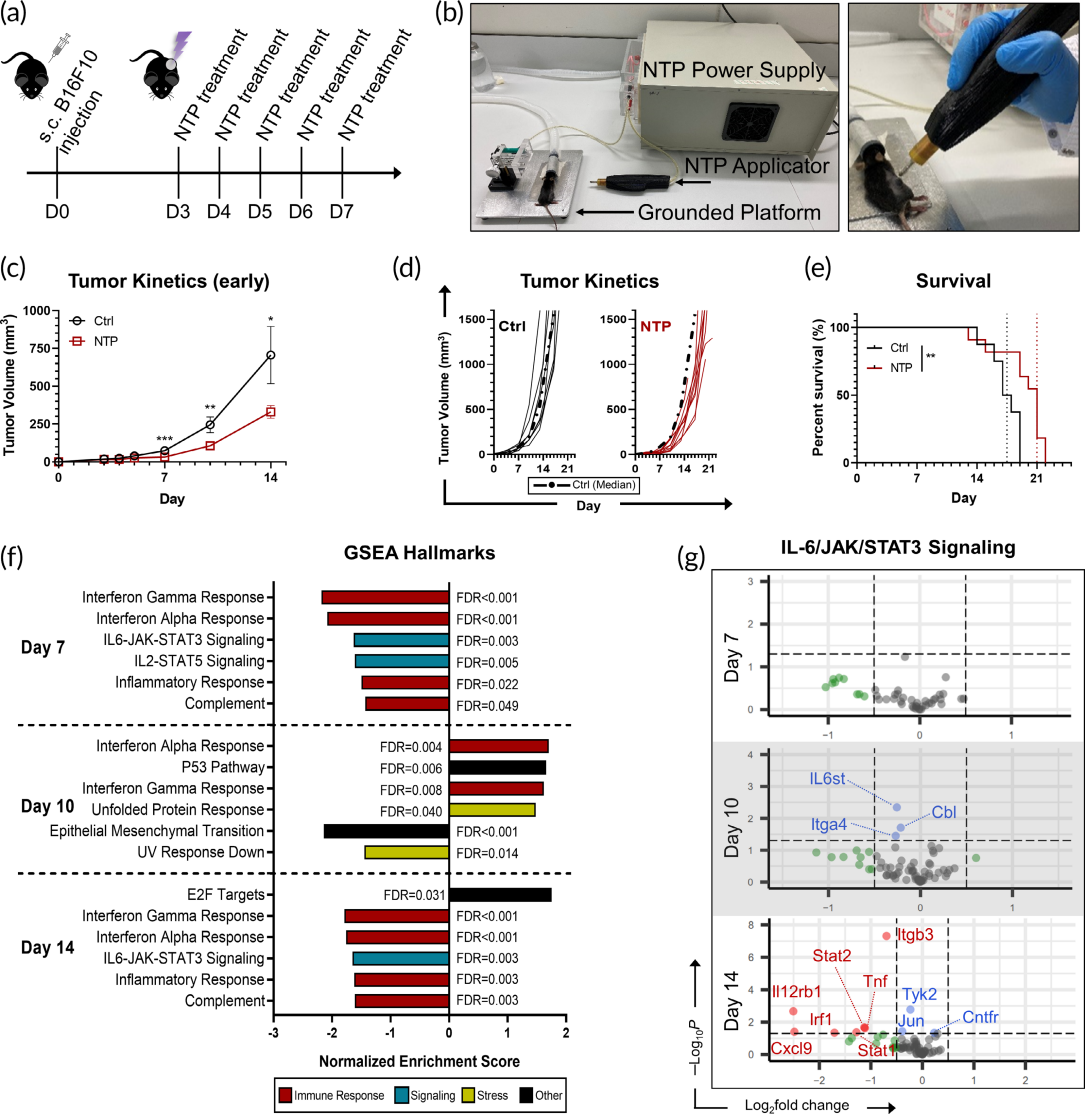
Anti‐cancer effects of NTP therapy. (a) Melanoma tumor‐bearing mice were treated with NTP for 5 consecutive days. (b) The NTP system consisting of a microsecond‐pulsed power supply and a DBD applicator was used to generate NTP directly onto the tumor surface. (c) Immediately after the fifth day of NTP treatment, tumor volumes were significantly smaller compared to untreated controls (Ctrl). Data shown are mean ± SEM from two independent experiments (Ctrl, *n* = 8; NTP, *n* = 11). *p≤ 0.05, **p ≤ 0.01, ***p ≤ 0.001 (generalized linear mixed model). (d) Tumor kinetics were monitored until humane endpoints were reached, and (e) Kaplan–Meier plots showed a significant increase in median survival in the NTP‐treated group. **p ≤ 0.01. Tumors were resected immediately after the last treatment (Day 7) or 3 (Day 10) or 7 (Day 14) days post. RNA sequencing and transcriptome profiling were performed using a preranked GSEA. (f) Several significantly (FDR ≤ 0.05) enriched pathways are shown. The IL‐6/JAK/STAT3 was of particular interest, and (g) the top downregulated genes in the pathway from DEseq2 analysis are shown. Dotted lines indicate thresholds for uncorrected *p* ≤ 0.05 (*y* axis) and log2fold change ≤−0.5 or ≥0.5 (*x* axis). Genes labeled in blue or red are above the uncorrected *p* value threshold (based on log2fold change threshold), while points in green (above log2fold change threshold) are not

To determine the anti‐tumor efficacy of NTP therapy, B16F10 melanoma cells were cultured in vitro and subcutaneously injected into syngeneic C57BL/6J mice to establish tumors. Once tumors became palpable (17.8 ± 2.5 mm^3^; mean ± SEM), they were treated locally with NTP (Figure 2a). Treatment was performed once per day (Figure 2b) for 5 consecutive days, and tumor kinetics and animal survival were monitored until humane endpoints were reached. NTP significantly reduced tumor volumes compared to the control (Figure 2c), as early as the last day of the treatment regimen (Day 7: 32.2 ± 5.8 mm^3^ vs. 74.1 ± 9.4 mm^3^; *p* = 0.0009). The tumor burden was still significantly reduced (*p* = 0.0472) on Day 14 (330.8 ± 41.9 mm^3^) compared to that of the untreated control (706.6 ± 189.7 mm^3^). While NTP therapy had a significant effect on the tumor volume initially and even 7 days after the final treatment (Figure 2c), the growth kinetics of the tumor in each mouse appeared unchanged once treatment was stopped (Figure 2d). Still, NTP treatment improved mouse survival, as the median survival of the mice receiving treatment was extended by 3.5 days (*p* = 0.0086) (Figure 2e). Necropsy performed immediately after humane endpoints were reached, did not reveal any metastatic lesions in the mice of either untreated or NTP‐treated groups. Altogether, these data evidence the anti‐cancer potential of our NTP treatment scheme and suggests that the direct effects of NTP on the tumor occur locally and early after treatment. Further analysis of the NTP effects on the tumor and subsequent immune responses were performed using this treatment schedule.

To gain broad insight into the molecular effects of NTP treatment, tumors were resected immediately (Day 7), 3 days after (Day 10), or 7 days after (Day 14) the last day of treatment and subjected to whole transcriptome sequencing (RNA sequencing). A preranked (based on a significance score described in Section 5, ^34^) Gene Set Enrichment Analysis (GSEA) was performed to evaluate temporal changes to the tumor profile following NTP treatment. Several signaling pathways were downregulated at the end of NTP treatment, which persisted into Day 14 (Figure 2f).

Of particular interest is the IL‐6/JAK/STAT3 pathway, which is known to drive tumor proliferation, survival, and even metastasis.^35^ This pathway is hyperactive in many types of cancers, including melanoma, and hyperactivation of STAT3 in tumor‐infiltrating immune cells is associated with an immunosuppressive effect.^36^, ^37^ Here, the IL‐6/JAK/STAT3 pathway was downregulated on Day 7 (NES = −1.65, FDR = 0.003) and Day 14 (NES = −1.63, FDR = 0.003), suggesting that modulation of this pathway is partially responsible for the observed NTP effects on tumor kinetics and mouse survival (Figure 2g). Another gene set of interest is the unfolded protein response (UPR), which was upregulated across all days, though only significantly on Day 10 (NES = 1.48, FDR = 0.040) (Figure 2f). The UPR is a signaling pathway that is stimulated during endoplasmic reticulum (ER) stress, and ER‐stress mediated cell death is tightly associated with the release of danger signals characteristic of ICD.^38^ These results are in‐line with our previous report that NTP induces ICD in melanoma cells in vitro.^13^ The induction of ICD should stimulate an anti‐cancer immune response, and interestingly, we see dynamic changes in immunological pathways, such as interferon gamma (IFN‐γ) and alpha (IFN‐α), with an increase on Day 10 (IFN‐γ: NES = 1.62, FDR = 0.008; IFN‐α: NES = 1.70, FDR = 0.004). A detailed list of the core enrichment genes and analysis scores and the genes from the differential gene expression (DESeq2) analysis are also provided (Tables S1 and S2, respectively).

Taken together, our data suggest that NTP could reduce tumor burden and prolong mouse survival via modulating the tumor profile and microenvironment and stimulating a dynamic anti‐cancer immune response. Therefore, in the next sections, we investigated how NTP treatment affects tumor immunogenicity and stimulates the anti‐cancer immunity cycle over time (Days 7, 10, and 14).

### 2.3 NTP affects tumor immunogenicity

**FIGURE 3:**
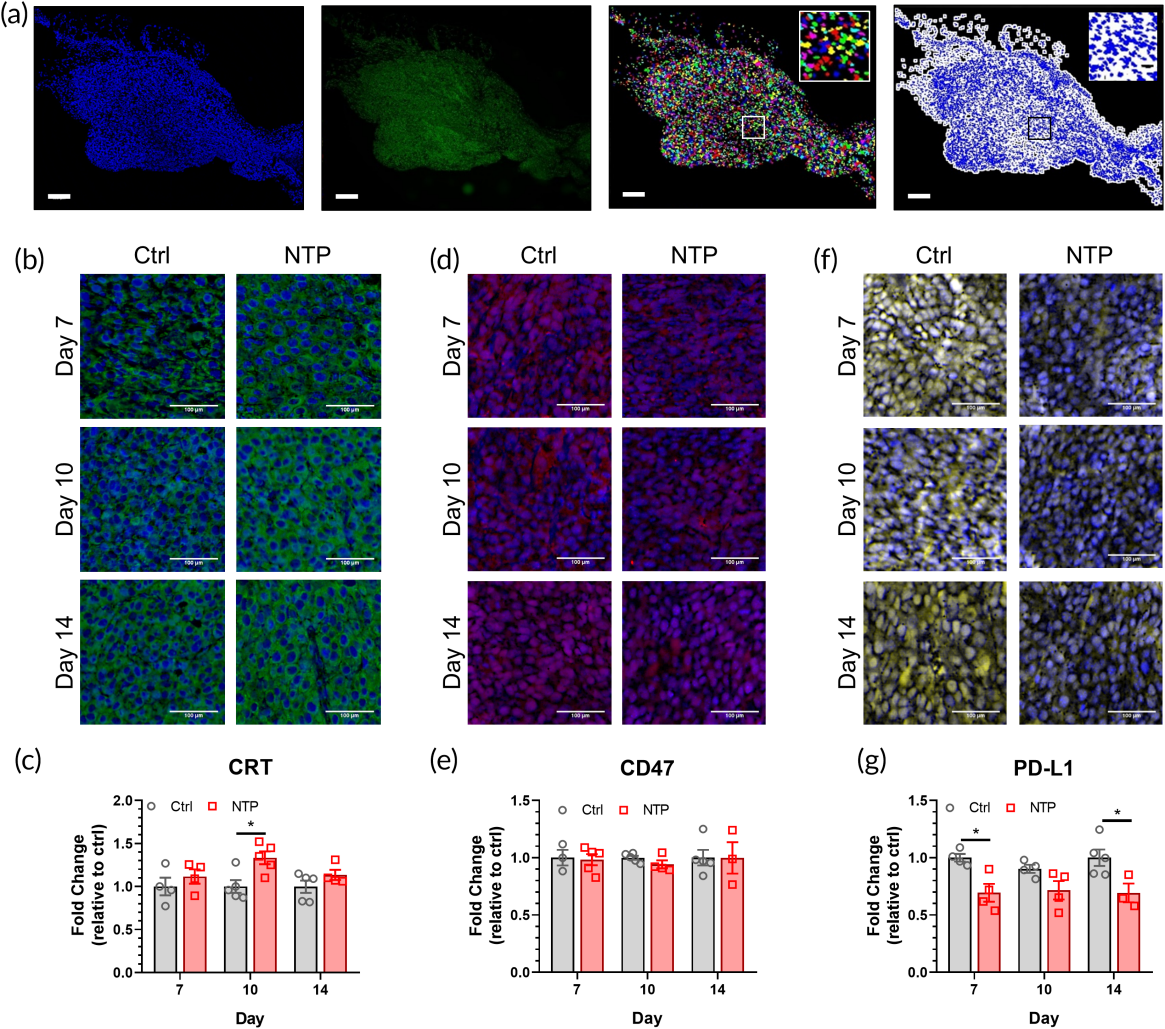
NTP treatment affects tumor immunogenicity. Tumor sections were collected on Days 7, 10, and 14, individually stained for specific cancer proteins, and counterstained with DAPI to identify the nuclei. (a) Quantification of protein expression was performed with our software, which identifies cell nuclei from the DAPI stain (far left panel; blue) within the tumor and analyzes the signal around it (middle left panel; green). A representative mask of the nuclei is shown (middle right panel) along with the analysis area (far right panel; white). Inserts are 500 μm × 500 μm zoomed‐in sections to show the segmentation capability of the software and analysis. All scale bars are 500 μm. Representative images of the (b) CRT staining (green), (d) CD47 staining (red), and (f) PD‐L1 staining (yellow) are shown. Scale bars are 100 μm. The immunofluorescence signal was quantified with our program and graphed for (c) CRT, (e) CD47, and (g) PD‐L1. Data are represented as mean ± SEM and each biologically independent sample is shown (*n* = 3–5). Statistical significance of the NTP treatment condition was compared to that of the control for each day, using the generalized linear mixed model. *p ≤ 0.05* *  (detailed *p* values are reported in the text)

Given that NTP has been reported to induce ICD, and an increase in the UPR pathway was measured in the transcriptome analysis (Figure 2f), we evaluated how NTP affects tumor immunogenicity. Tumors were resected, sectioned, and stained individually for specific cancer ligands. Quantification was performed with our developed software, which identifies cell nuclei within the tumor and analyzes the signal around it (Figure 3a).

The expression of calreticulin (CRT) and CD47 in the tumor was evaluated, as they are key signals for the innate immune response. CRT is a hallmark of ICD and a potent pro‐phagocytic signal that is counterbalanced by CD47, an anti‐phagocytic signal.^39^ Following treatment, CRT expression increased slightly on Day 7 and significantly on Day 10 (1.33 ± 0.07 fold; *p* = 0.0128) (Figure 3b,c), while no significant change was observed in CD47 (Figure 3d,e). By Day 14, CRT expression was returning to baseline for the NTP treatment group (1.15 ± 0.076; *p* = 0.1969), thus highlighting the transient effect of NTP. This corresponded with the GSEA, which also showed the highest UPR activation on Day 10 (Figure 2f).

The expression of programmed death‐ligand 1 (PD‐L1), a critical checkpoint for the adaptive immune response, was also evaluated. Following NTP treatment, PD‐L1 expression was immediately reduced (0.69 ± 0.08; *p* = 0.011), and interestingly, remained low on Day 10 (0.72 ± 0.08; *p* = 0.078) and 14 (0.69 ± 0.08; *p* = 0.034) (Figure 3f,g). This indicates that NTP may have persistent effects on PD‐L1 expression.

Taken together, the data indicate that NTP treatment modulates the expression of cellular proteins in the tumor, and the durability of the NTP effect seems transient for some proteins while it is persistent for others.

### 2.4 NTP affects dendritic cells and antigen presentation

**FIGURE 4:**
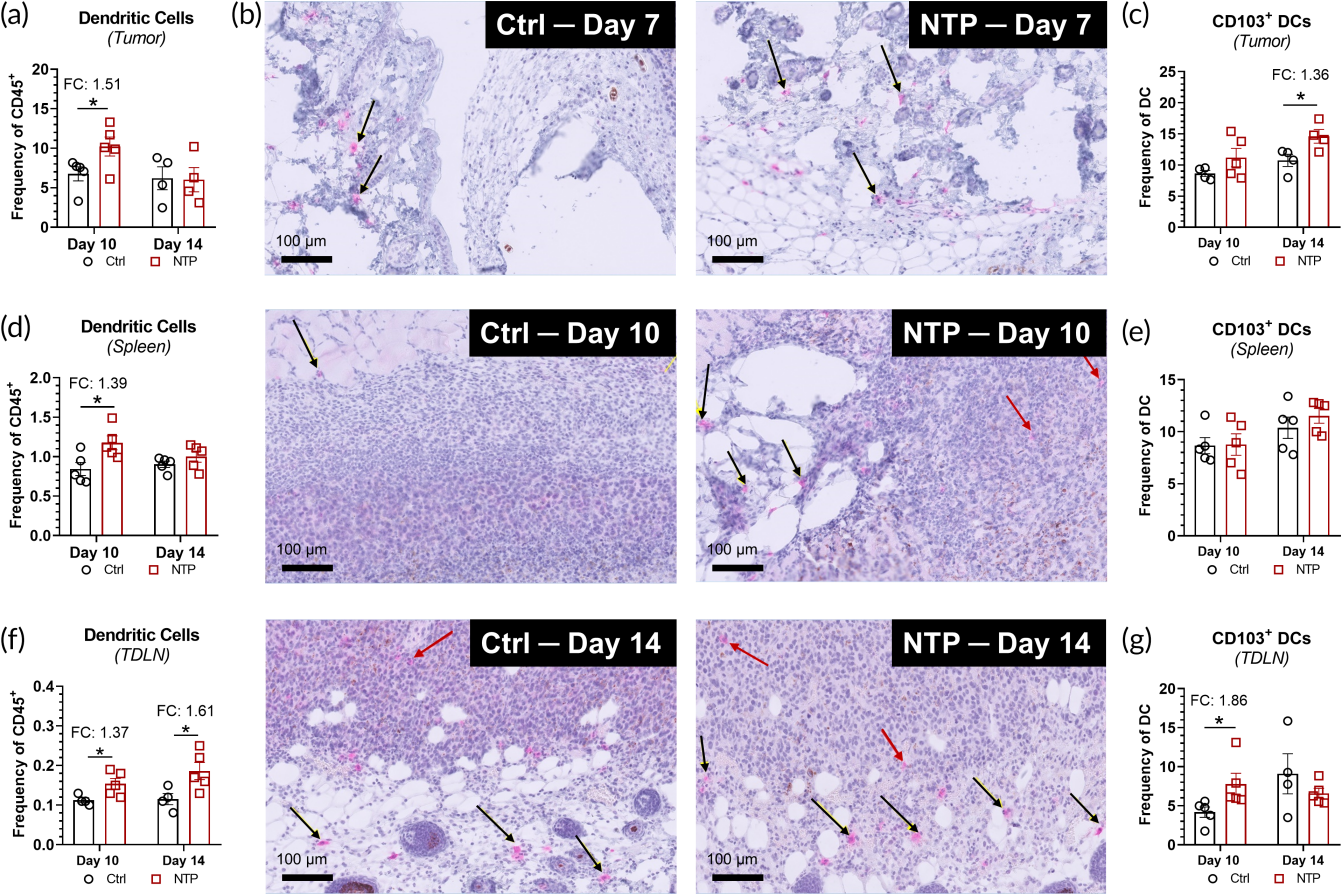
NTP treatment increases DCs in the tumor and surrounding lymph organs. (a) Flow cytometry analysis showed a higher frequency of DCs (MHC‐II^+^/CD11c^+^) in the tumor on Day 10. (b) IHC analysis of tumor sections on Days 7, 10, and 14 also showed higher amount of CD11c^+^ antigen‐presenting cells, with localization occurring predominantly in the periphery of the tumor (black arrows) compared to the center (red arrows). c) CD103^+^ DCs, a subtype responsible for cross‐presentation of antigens to T cells, were also measured in the tumor. Flow cytometry analysis of the spleen (d, e) and TDLN (f, g) also showed a higher frequency of DCs and CD103^+^DCs following NTP treatment. Data are represented as mean ± SEM and each biologically independent sample is shown (*n* = 3–5). Statistical significance was determined using the generalized linear mixed model and the NTP treatment condition was compared to that of the control for each day. The fold change (FC) compared to the untreated control is also reported for statistically significant comparisons. *p ≤ 0.05 (detailed *p* values are reported in the text)

Following an increase in tumor immunogenicity, it is crucial for antigen‐presenting cells to be recruited and activated for the development of specific anti‐cancer immunity.^28^ Dendritic cells (DCs), which are key antigen‐presenting cells, must capture tumor antigens from dead and dying cancer cells for processing and antigen presentation.^40^ Therefore, we evaluated the presence of DCs (MHC‐II^+^/CD11c^+^) in the tumors using flow cytometry analysis. The detailed gating strategy is provided in supplementary information (Figure S4). Since tumors were too small for analysis immediately at the end of NTP treatment (Day 7), analysis was performed 3 and 7 days after the last NTP treatment, on Days 10 and 14, respectively. On Day 10, the intratumoral DCs were increased 1.51‐fold (*p* = 0.0489) following NTP treatment compared to the untreated control (Figure 4a). Tumors sections were also stained for CD11c as a marker for antigen‐presenting cells following resection on Days 7, 10, and 14. Each slide was scored by a pathologist on the density of CD11c^+^ cells in the periphery and center of the tumor, with 0 being empty to 3 being dense, and the mean density was reported (Table 2). Based on the evaluation, tumors treated with NTP showed a higher density of CD11c^+^ cells compared to the untreated, and localization occurred predominantly in the periphery (Figure 4b).

**TABLE 2:** Histological scoring of immune cell populations in the tumor

		CD11c^+^		CD8^+^ T cells		CD4^+^ T cells	
	*Group*	*Periphery*	*Tumor center*	*Periphery*	*Tumor center*	*Periphery*	*Tumor center*
Day 7	Ctrl	1.0^a^	0.0	0.0	0.0	2.0	0.5^b^
	NTP	1.4^b^	0.2	0.0	0.0	2.0	0.0
Day 10	Ctrl	1.8^b^	0.6	0.2	0.0	2.6^b^	0.4
	NTP	2.0^a^	1.3	0.4	0.2	2.0	0.0
Day 14	Ctrl	1.6^b^	1.0	0.2	0.0	2.8^b^	0.0
	NTP	2.8^b^	1.0	0.0	0.0	3.0^a^	0.8

*Note*: 0 empty; 1 sparse; 2 moderate; 3 dense.

^a^ Value observed in 100% of samples.

^b^ Value  observed in ≥ 50% of samples.

CD103^+^ DCs, a subtype with high capacity for antigen cross‐presentation to T cells,^41^ were also quantified in the tumor with flow cytometry to inform on the antigen presentation stage of the cancer‐immunity cycle. A slight increase was observed on Day 10 in the NTP treatment group (11.2 ± 1.5%) compared to untreated (8.6 ± 0.5%), though the results were not statistically significant (*p* = 0.1795). However, by Day 14, the percentage of CD103^+^ DCs in the NTP treatment group significantly increased compared to untreated (1.36‐fold increase; *p* = 0.0388) (Figure 4c).

After capturing tumor antigens in the tumor, DCs must migrate to the surrounding lymph organs (spleen or TDLN) for antigen presentation to specific T cells.^42^, ^43^ This is a crucial step for activation of anti‐cancer adaptive immunity, and therefore, we also investigated the presence of DCs and CD103^+^ DCs in the spleen and TDLN. On Day 10, we observed a significant increase in DCs in both the spleen (1.39‐fold; *p* = 0.0258) and the TDLN (1.37‐fold; *p* = 0.0394) for the NTP treatment group (Figure 4d,f). The DC population increased further by Day 14 (1.61‐fold; *p* = 0.0368) in the TDLN (Figure 4f **)**. When investigating whether these DCs are more primed for cross‐presentation, no significant difference in the CD103^+^ DCs was observed in the spleen (Figure 4e). However, a 1.86‐fold increase in the NTP treatment group (*p* = 0.0476) was measured in the TDLN on Day 10 (Figure 4g). Therefore, it appears that NTP treatment enhanced the DCs in both lymph organs, and the increase in DCs persisted longer in the TDLN. Furthermore, the crucial subset of DCs that bridges the innate and adaptive anti‐tumor immunity was increased in the lymph node on Day 10.

Taken together, the data suggest that NTP treatment can enhance anti‐cancer immunity by increasing antigen‐presenting cells to the tumor (primarily in the periphery) and enhancing their migration to the surrounding lymph organs for antigen presentation.

### 2.5 NTP affects T cells in the tumor‐draining lymph node

Presentation of captured tumor antigens from DCs must result in the priming and activation of T cells in order for the anti‐cancer immunity cycle to progress.^28^ Therefore, we evaluated the cytotoxic (CD8^+^) and helper (CD4^+^) T‐cell populations in both the spleen and lymph node after NTP treatment. Using FoxP3 and CD25 lineage markers, CD4^+^ T cells were further discriminated between T regulatory cells (Tregs; FOXP3^+^/CD25^+^) and non‐Tregs (FOXP3^−^/CD25^−^). A detailed gating strategy is provided in supplementary information (Figure S5).

**FIGURE 5:**
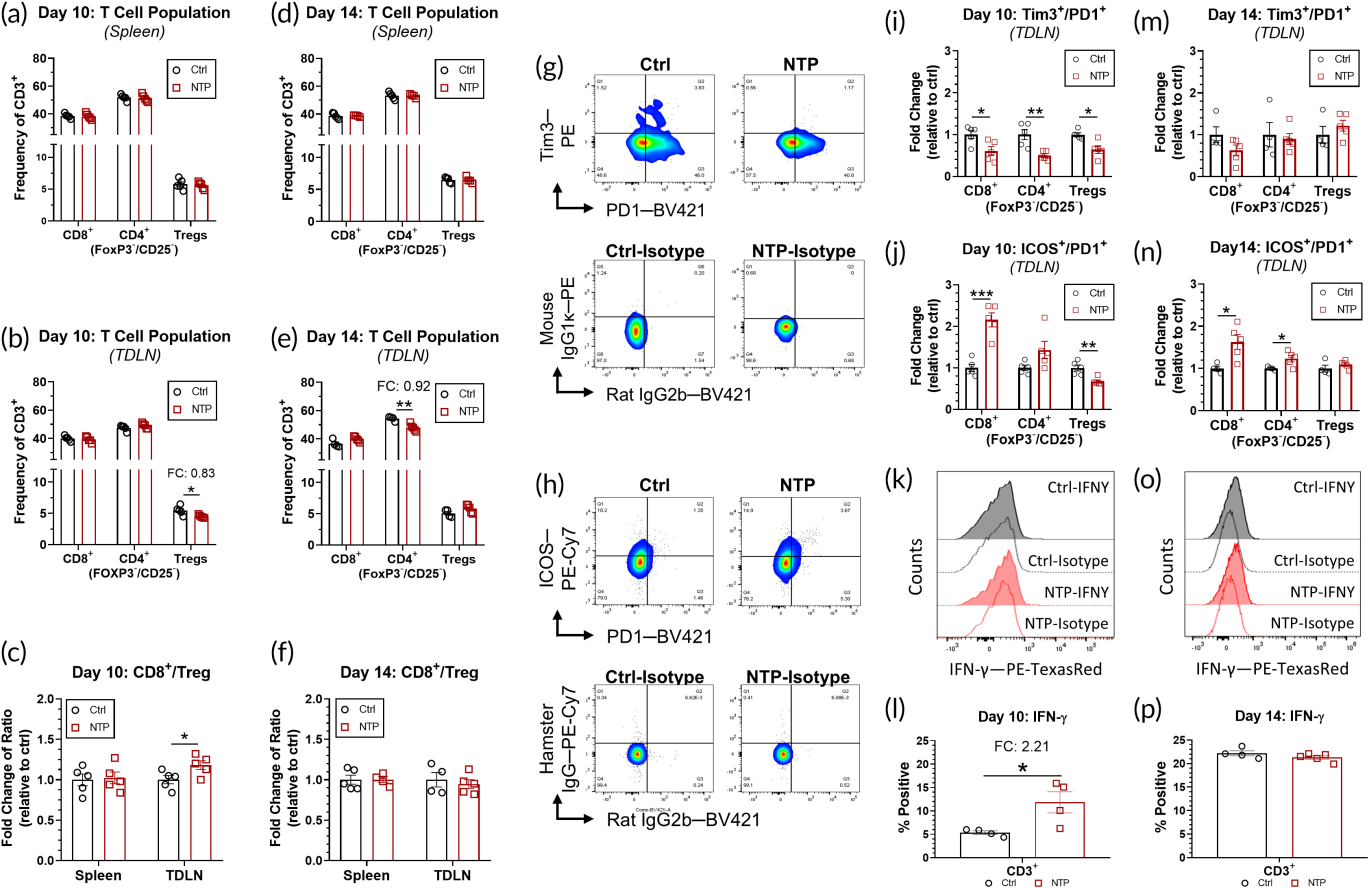
NTP treatment affects T‐cell status in the tumor‐draining lymph node. CD8^+^ T cells, non‐regulatory CD4^+^ T cells (FOXP3^−^/CD25^−^), and Tregs (FOXP3^+^/CD25^+^) were measured on Day 10 in the (a) spleen and (b) TDLN, and the (c) ratio of cytotoxic CD8^+^ T cells and immunosuppressive Tregs was calculated. (d)–(f) By Day 14, no changes were observed between the groups. The T‐cell status was also measured using flow cytometry to further investigate changes in the TDLN T‐cell populations. (g) T cells were dual stained for Tim3 and PD1 as an indicator of T‐cell exhaustion (Tim3^+^/PD1^+^). Representative dot plots of the control and NTP‐treated groups are shown and gating was established based on the corresponding isotype controls. Quantification of Tim3^+^/PD1^+^ populations of the 3 T‐cell subtypes showed less exhaustion in the NTP‐treatment group compared to the control on (i) Day 10, which returned to baseline by (m) Day 14. (h) Non‐exhausted ICOS^+^/PD1^+^ T cells were gated and analyzed in a similar fashion as an indicator of T‐cell activation. Quantification showed lower Treg activation on (j) Day 10, and higher activation of CD8^+^and non‐regulatory CD4^+^ T cells on both (j) Day 10 and (n) Day 14. CD3^+^ T‐cell production of IFN‐γ was also analyzed. (k, o) Representative histograms are shown, and overton quantification of the percent positive cells revealed an increase in CD3^+^ IFN‐γ expression in the NTP‐treatment group compared to the control on (l) Day 10. (p) By Day 14, IFN‐γ expression was equivalent in both groups. Data are represented as mean ± SEM and each biologically independent sample is shown (*n* = 4–5). Statistical significance was determined using the generalized linear mixed model and the NTP treatment condition was compared to that of the control for each day. The fold change (FC) compared to the untreated control was also reported for statistically significant comparisons. *p ≤ 0.05, **p ≤ 0.01, *** *p* ≤ 0.001 (detailed *p* values are reported in the text)

No difference was observed in the CD8^+^ or CD4^+^(FOXP3^−^/CD25^−^) T cell populations between the control or NTP treatment groups on Day 10 in both the spleen (Figure 5a) and the TDLN (Figure 5b). While these T cells perform anti‐tumor functions, Tregs are known to be immunosuppressive and can impair prognosis by downregulating effector T cell activation. Interestingly, Treg population in the lymph node was reduced 3 days following NTP treatment (Day 10: 0.83‐fold change; *p* = 0.0246) (Figure 5b). The ratio of cytotoxic T cells to Tregs (CD8^+^/Treg) provides a crucial balance for a strong anti‐tumor immune response.^44^ The decreased Treg population resulted in a higher CD8^+^/Treg ratio in the NTP treatment group (1.18‐fold increase; *p* = 0.0414), thus pointing to a stronger activating, anti‐cancer immune response (Figure 5c). By Day 14, all T‐cell populations were similar between the two groups in both the spleen (Figure 5d) and TDLN (Figure 5e), except for a slight decrease in the CD4^+^ (FoxP3^−^/CD25^−^) population in the TDLN of the NTP group (*p* = 0.0038). The CD8^+^/Treg ratio was also the same (Figure 5f) suggesting that NTP affects the T‐cell population in the early stages of the cancer‐immunity cycle.

To further investigate the T‐cell population of NTP‐treated mice, we measured ICOS, PD1, IFN‐γ, and Tim3 expression as an indicator of T‐cell activation (ICOS^+^/PD1^+^, IFN‐γ) and exhaustion (Tim3^+^/PD1^+^). ICOS on T cells have been shown to have a crucial function in memory and effector T‐cell development, and interaction with its ligand is linked to the release of immunostimulatory and anti‐cancer cytokines.^45^, ^46^ However, it should be noted that while an increase in ICOS expression in CD8^+^ T cells and non‐regulatory CD4^+^ T cells (non‐Tregs) is desirable for anti‐cancer immunity, higher ICOS expression in Tregs has been shown to be more immunosuppressive compared to lower ICOS expressing Tregs. Indeed, that is why careful distinctions between Tregs and non‐Tregs (CD4^+^FOXP3^−^CD25^−^) were made in the analysis. A detailed gating strategy is shown in supplementary information (Figure S5) and quantification of exhausted or activated T cells was corrected by the corresponding isotype control (Figure 5g,h).

T‐cell exhaustion refers to a process where T cells gradually lose their function, and in the context of tumor development, cannot perform their anti‐cancer processes. Co‐expression of Tim3^+^/PD1^+^ on T cells has been shown to indicate deep exhaustion, and higher levels of Tim3 on CD8^+^ T cells have been associated with poor prognosis.^47^ Moreover, higher Tim3 expression on Tregs has been shown to enhance regulatory and immunosuppressive functions. Quantification of exhausted T cells with corresponding isotype control correction (Figure 5g) revealed less Tim3^+^/PD1^+^ T cells for all subpopulations compared to that of untreated controls on Day 10 (Figure 5i). Importantly, T cells do not appear more exhausted by day 14 following NTP treatment (Figure 5m).

On day 10, we not only observed a higher frequency of ICOS^+^/PD1^+^ expression in non‐exhausted, cytotoxic T cells (2.158‐fold change; *p* = 0.0003), but also a lower frequency in Tregs (0.672‐fold change; *p* = 0.0040), thus indicating higher activation (Figure 5j). Furthermore, IFN‐γ expression, a key cytokine in activating cellular immunity, was also increased in CD3^+^ immune cells on the same day. A rightward shift in the peak IFN‐γ fluorescence was observed (Figure 5k) and overton quantification of the percent positive cells showed a 2.21‐fold increase (*p* = 0.0303) in the NTP‐treated group (Figure 5l), though this response was dynamic (Figure 5o,p). Interestingly, the expression of ICOS^+^/PD1^+^ remained high for CD8^+^ (1.636‐fold increase; *p* = 0.0188) and non‐regulatory CD4^+^ T cells (1.234‐fold increase; *p* = 0.0352) (Figure 5n).

Therefore, it appears that following NTP treatment, adaptive immune cells are also stimulated to have a more anti‐cancer profile.

Taken together, it appears that NTP treatment can transiently potentiate the next steps of the cancer‐immunity cycle in the TDLN as more CD8^+^ and non‐regulatory CD4^+^ T‐cell activation was measured along with less exhaustion. This parallels the measured increase in IFN‐γ. Moreover, it has been reported that an increase in ICOS expression in both CD4 and CD8 T cells parallels an increase in the CD8^+^/Treg ratio.^45^ Indeed, while we did not observe statistically significant changes to the T‐cell subtype populations, the CD8^+^/Treg ratio was significantly higher in NTP‐treated mice (Figure 5c), which is advantageous for developing robust anti‐cancer immunity.

### 2.6 NTP affects T cells in the tumor and cytotoxic immune responses

**FIGURE 6:**
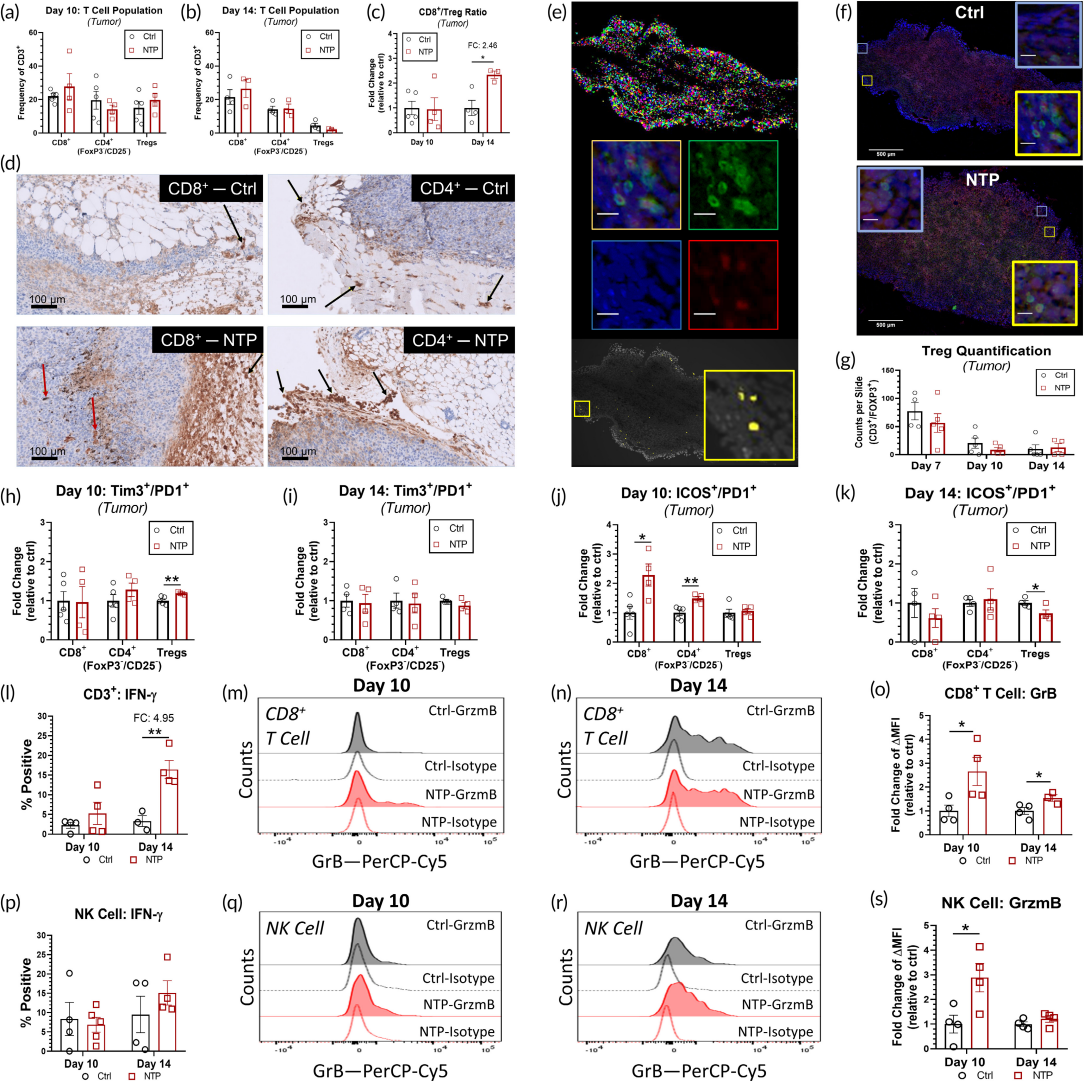
NTP treatment elicited more CD8^+^ and non‐regulatory CD4^+^ T‐cell activation in the tumor. Flow cytometry analysis revealed that NTP treatment did not significantly affect effector T‐cell populations on (a) Day 10 or b) Day 14. (c) However, the ratio of CD8^+^ T cells to Tregs was significantly increased on Day 14. Tumor sections were also (d) stained with IHC for CD8^+^ and CD4^+^ T cells. Scale bars are 100 μm, and red arrows indicate labeled T cells in the tumor center, while black arrows indicate labeled T cells in the tumor periphery. (e) Dual immunofluorescence staining for CD3 and FOXP3 was evaluated using our program to identify the Tregs in the tumor tissue. The top panel demonstrates the segmentation capability of the software to identify DAPI staining, from which a mask of double positive cells is made (bottom panel, in yellow) based on the fluorescence intensity of each channel (middle). (f) Representative images of the control and NTP‐treated tissue are shown and yellow inserts show examples of double positivity while blue boxes demonstrate cells that are negative. Scale bars in all inserts are 25 μm while scale bars in main images are 500 μm. (g) The number of Tregs were quantified for each tumor slide. Flow cytometry analysis of T cells utilized (h), (i) Tim3^+^/PD1^+^ as a marker for exhaustion and (j), (k) ICOS^+^/PD1^+^ as a marker for activation. (l) CD3^+^ cells in the NTP‐treated tumors exhibited higher IFN‐γ on day 14, while (p) no changes were observed in NK cells. Representative histograms of granzyme B (GrB) staining for (m, n) CD8^+^ T cells and (q, r) NK cells along with the corresponding isotypes are shown. Quantification of mean fluorescence intensity normalized to the isotype (∆MFI) showed higher o) cytotoxic T cell and (s) NK cell expression of GrB. Statistical significance was determined using the generalized linear mixed model and the NTP treatment cohort was compared to that of the untreated control for each day. The fold change (FC) was also reported for statistically significant comparisons. *p ≤ 0.05, **p ≤ 0.01 (detailed *p* values are reported in the text)

Following T‐cell activation, it is critical that the activated T cells are trafficked back to the tumor and infiltrate the tumor bed for specific recognition and killing of cancer cells. Therefore, the T‐cell populations in the tumor were also evaluated. No significant differences were measured in the T‐cell subpopulations on Day 10 or Day 14 (Figure 6a,b). However, interestingly, the ratio of CD8^+^ T cells to Tregs was significantly increased on day 14 (2.46‐fold; *p* = 0.0177) (Figure 6c). IHC staining for CD8^+^ and CD4^+^ T cells was also performed on tumor sections (Figure 6d) resected on Days 7, 10, and 14 and scoring by a pathologist also led to similar results (Table 2). Dual immunofluorescence stainings of CD3 and FOXP3 on the tumor sections were analyzed for the number of Tregs (CD3^+^/FOXP3^+^) per tumor section using our developed software (Figure 6e). Representative images of Tregs are shown (Figure 6f), and quantification revealed that the number of Tregs was equivalent between the NTP‐treated and untreated control groups (Figure 6g), which further corroborated our flow cytometry analysis (Figure 6a,b).

An in‐depth look into the intratumor T‐cell state revealed no significant difference in Tim3^+^/PD1^+^ CD8^+^ and non‐regulatory CD4^+^ T cells on either day and only a slight increase in the Treg population on Day 10 (Figure 6h, i). However, the CD8^+^ and non‐regulatory CD4^+^ T cells expressed more ICOS^+^/PD1^+^ on Day 10 compared to untreated (2.29‐fold; *p* = 0.0171 and 1.48‐fold; *p* = 0.0073, respectively) (Figure 6j). While this activation marker returned to baseline on Day 14 for CD8^+^ and non‐regulatory CD4^+^ T cells, dual expression of ICOS^+^/PD1^+^ Tregs was reduced 0.73‐fold (*p* = 0.0474) (Figure 6k), which also indicates a more immunostimulatory tumor microenvironment. Furthermore, the expression of IFN‐γ in CD3^+^ cells was higher (4.95‐fold; *p* = 0.0066) compared to controls (Figure 6l). These data indicate that NTP treatment elicits a more immunostimulatory anti‐cancer immune response profile, which persists to Day 14, as shown by higher IFN‐γ in CD3^+^ cells, lower ICOS^+^/PD1^+^ Tregs, and a higher CD8^+^/Treg ratio.

Natural killer (NK) cells are another subset of lymphocytes that can participate in shaping adaptive anti‐cancer immune responses through the secretion of cytokines such as IFN‐γ, though they are most notable for their ability to specifically identify and kill cancerous cells. Together with cytotoxic T cells, NK cells can directly kill tumor cells via the perforin and granzyme system. Following NTP treatment, we did not observe an increase in NK cell population in either the tumor or TDLN (Figure S6), and IFN‐γ levels were also equivalent with the that of the controls (Figure 6p). However, interestingly, while the CD8^+^ T cells and NK cell frequency in the tumor remained unchanged, the production of granzyme B (GrB) was elevated in both lymphocyte CD8^+^ T cells (Figure 6m–o) and NK cells (Figure 6q‐s). The mean fluorescence intensity of GrB was quantified following isotype correction (∆MFI) and revealed a 2.65‐fold increase (*p* = 0.0406) for CD8^+^ T cells (Figure 6o) and a 2.89‐fold increase (*p* = 0.0322) for NK cells on Day 10 (Figure 6s). Furthermore, GrB expression in CD8^+^ T cells remained elevated (1.54‐fold; *p* = 0.0470) on Day 14.

Taken together, though NTP treatment did not significantly affect T cell or NK cell populations in the tumor, it appears to enhance the activation of the T cells present, and the cytotoxic activity of both CD8^+^ T cells and NK cells. Along with the observed increase in the CD8^+^ T cell to Treg ratio, these data suggest that NTP treatment is modulating the tumor microenvironment and enhancing anti‐cancer immunity.

## 3 DISCUSSION

**FIGURE 7:**
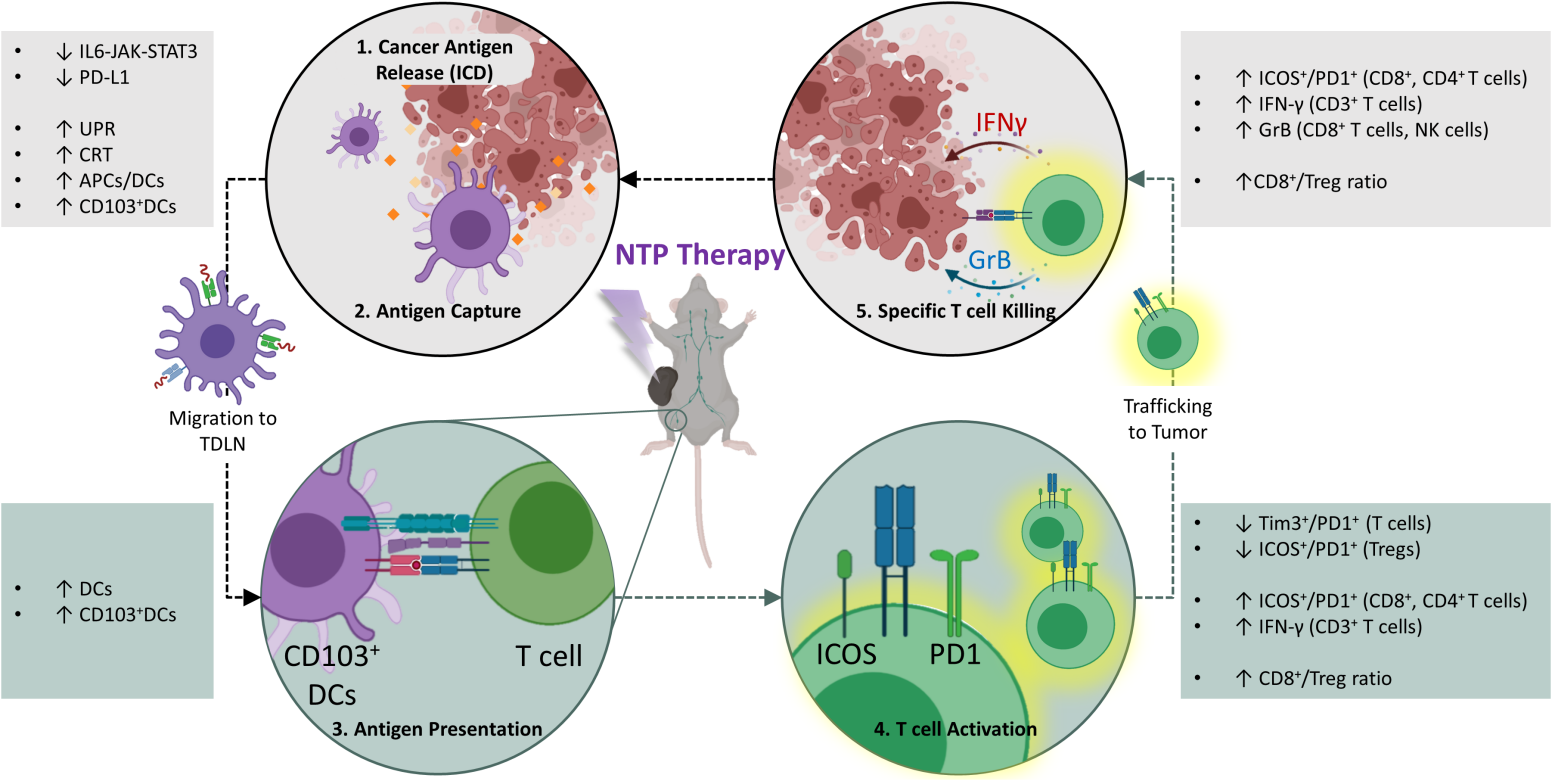
The cancer‐immunity cycle in response to NTP therapy

Preclinical and clinical reports indicate that NTP is a well‐tolerated therapy and holds great promise as an adjuvant therapy for melanoma, but before this technology can be further developed for clinical use, a detailed understanding of how NTP treatment of tumors affects the cancer‐immunity cycle is urgently needed. Here, we addressed this by investigating the tumoral and immunological effects of a local NTP therapy in melanoma tumor‐bearing mice. The various effects of NTP observed in our experiments are summarized for different stages of the cancer‐immunity cycle (Figure 7).

Based on RNA sequencing analysis of the tumor, we observed the signaling pathway most affected by NTP was the IL‐6/JAK/STAT3 pathway, which was downregulated after treatment. This is highly favorable, as hyperactivation of this pathway has been associated with tumor progression, immunosuppression, and poor clinical prognosis and therefore, warrants independent follow‐up studies to validate these findings. In‐line with previous in vitro and in vivo reports,^12^, ^13^, ^14^, ^15^, ^18^ our NTP treatment also induced acute UPR activation and CRT emission, which are characteristic of ICD in the tumor.^48^, ^49^ This was followed by higher infiltration of DCs into the tumor and more migration to the spleen and TDLN. Moreover, a higher frequency of CD103^+^ DCs was observed in both the tumor and TDLN of mice receiving NTP treatment, which indicates more DCs are primed for antigen presentation to T cells.^41^ Interestingly, while the number of T cells remained unchanged in the tumor and lymph organs, those found in the tumor and TDLN following NTP treatment expressed more activation (ICOS^+^/PD1^+^, IFN‐γ) and less exhaustion (Tim3^+^/PD1^+^) markers. Immune cells in the NTP‐treated mice also had higher cytotoxic capacity in the tumor, as indicated by increased GrB expression in both CD8^+^ T cells and NK cells. The effect of NTP on other cell types within the tumor has also been previously reported (e.g., cancer‐associated fibroblasts,^50^ tumor‐associated macrophages^51^) and will be investigated in future studies. Furthermore, it is of great interest to determine the localization of recruited immune cells in the tumor microenvironment with respect to the site of NTP treatment.

Taken together, our data indicate that local application of NTP on subcutaneous melanoma, can affect tumor development pathways, promote ICD, and activate systemic, anti‐cancer immune responses. As different cancer types have varying levels of immunogenicity and responses to therapy, these experiments should also be performed in other cancer models. Indeed, there is building evidence of anti‐cancer immune activation following NTP therapy in different cancer types,^12^, ^15^, ^52^ and similar in‐depth investigation into the degree of stimulation and progression of the cancer‐immunity cycle should also be performed. Furthermore, several studies have also suggested that NTP therapy of primary tumors may have an effect at distant, untreated tumor sites.^15^, ^53^, ^54^ While we did not observe any metastatic lesions in any of our tumor‐bearing mice (treated or untreated), more sophisticated models^55^ will need to be used to fully evaluate NTP effect on secondary and metastatic tumors. These promising responses to NTP treatment require more detailed studies into not only the underlying mechanisms, but also into how NTP can be controllably dosed to bring about a predictable therapeutic response. Altogether, these fundamental insights can help direct the translation of NTP technology into the clinic and inform potential combination strategies for enhanced patient outcome.

In fact, in recent years, there has been an increased endeavor to find optimal combination strategies against tumors, using NTP in combination with existing therapies.^12^, ^52^, ^54^, ^56^, ^57^, ^58^, ^59^, ^60^ However, the possibility to combine NTP with promising therapeutics dramatically outnumber the resources, time, and eventually patients available, so combinations must be thoughtfully made to maximize benefits while minimizing resources. Peepers et al. have proposed several principles to develop rational cooperative or synergistic combination strategies,^61^ and our results can be applied to those principles, particularly (1) targeting drug‐resistant cell pools and signaling pathway reactivation, (2) modulating the tumor and microenvironment, and (3) sensitizing tumor cells to immunotherapy.

Melanoma is highly heterogeneous, and subclonal regions with phenotypic alterations can develop, resulting in certain drug‐resistant cell pools. Therefore, combining therapies that can target different drug‐resistant cell pools is an ongoing strategy. Radiotherapy has long been used for the management of melanoma, though melanomas have been historically considered to be radioresistant.^62^ Recently Pasqual‐Melo et al. demonstrated that NTP treatment sensitized melanoma cells to radiotherapy in vitro.^59^ Fractionated x‐ray therapy in combination with NTP induced higher apoptosis and cell cycle arrest. Furthermore, Sagwal et al. showed that combining NTP with several chemotherapeutics resulted in synergistic (doxorubicin and epirubicin) and additive (oxaliplatin) cytotoxicity against melanoma cells.^63^ Taken together, these studies suggest that NTP can be combined with radiotherapy and chemotherapy to target tumor regions with acquired resistance. Another approach to deal with therapy resistance is to reactivate previously targeted signaling pathways. Targeted therapies, such as BRAF inhibitors, have shown great clinical success against melanomas, but over time, cancerous cells can develop compensatory pathways, resulting in therapy resistance. Therefore, sensitizing these cells with therapies that reactivate the targeted signaling pathway is of particular interest. In fact, high secretion of IL‐6 has been associated with increased invasive migration in BRAF inhibitor‐resistant melanoma.^64^, ^65^ Furthermore, inhibition of STAT3 is currently under investigation to overcome acquired BRAF inhibitor resistance.^66^ As our whole transcriptome analysis indicates a decrease in the IL‐6/JAK/STAT3 pathway following NTP treatment, it stands to reason that combination with BRAF inhibitors could counteract acquired resistance by reactivating previous inhibitory pathways. A more in‐depth investigation into the activated and deactivated pathways following NTP treatment would be required to understand and leverage this combination strategy further.

Combining therapies that modulate the tumor and TME is also of high interest, as it is becoming more established that the complex interaction of the tumor with its local ecosystem contributes to drug resistance.^67^, ^68^ Melanoma cells are known to induce an immunosuppressive environment, but here in our study, we showed that NTP treatment modulated the tumor by increasing CRT and decreasing PD‐L1 expression on cancerous cells. Previous studies have reported that NTP treatment can oxidize proteins on the cancerous cell surface (e.g., CD47, CD44), which may interrupt tumor survival or immune evasion.^33^, ^69^ These appear effective when NTP is in direct contact with the cancerous cells and modifications occur rapidly. Interestingly, in our study, we observed a prolonged decrease in PD‐L1, which suggests that NTP can induce secondary signaling in addition to direct oxidation of surface proteins. Altogether, the capacity of NTP to modulate surface‐markers on tumor cells is intriguing and warrants further study, as this could be utilized when designing combination strategies.

Not only did our NTP treatment modulate the tumor, but it also recruited more DCs into the TME, and the anti‐cancer T cells were more activated and less exhaustive. This is highly desirable as the immunosuppressive environment induced by melanoma is known to elicit cytotoxic lymphocyte dysfunction.^70^ In fact, recent breakthroughs with immune checkpoint inhibitors, including anti‐PD‐1 and anti‐CTLA‐4, aim to restore CD8^+^ T‐cell function, though systemic administration of these inhibitors is often associated with heavy adverse effects. In our study, not only were more activated CD8^+^ and non‐regulatory CD4^+^ T cells found in the TME following local NTP treatment, but these activated cells were also found in the TDLN. Furthermore, the CD8^+^ T cells and the NK cells in the TME were modulated to exhibit a higher cytotoxic capacity, as indicated by elevated GrB levels. To our knowledge, there is currently only one report on how NTP‐treated cancer cells affect NK cell function, as investigation into this topic has only just begun. Clemen et al. demonstrated that co‐culturing NTP‐treated skin tumor cells with NK cells in vitro, enhanced the anti‐cancer functions of NK‐cells, including higher secretion of GrB.^71^ Taken together, the ability of NTP to stimulate anti‐tumor functions of T cells and NK cells in the TME is advantageous, though elucidation of the mechanism is still needed.

Another crucial non‐malignant cell type of the TME is the cancer‐associated fibroblast (CAF), which has been implicated to be particularly detrimental for the outcome of melanoma therapy.^70^ CAFs are highly involved in tumor angiogenesis, remodeling of the extracellular matrix, and secretion of pro‐tumorigenic and immunosuppressive factors.^70^ Indeed, STAT3‐associated resistance against BRAF‐inhibitors has been closely tied to the secretion of fibroblast growth factor 2 (FGF2) signaling.^66^ In addition, CAFs also present a physical barrier around the tumor, which can impede therapy. The effect of NTP on CAFs has only recently been proposed and studied.^19^, ^72^ Van Loenhout et al. reported that exposure to an NTP‐enriched solution eliminated pancreatic stellate cells (CAFs associated with pancreatic ductal adenocarcinoma) and modified them toward a more immunostimulatory profile.^19^ While the study was in the context of pancreatic cancer, the results were promising, and NTP effects on CAFs should also be investigated in melanoma.

In light of the recent breakthroughs with immunotherapies, another rational combination strategy is one that further sensitizes tumor cells to immunotherapy. So far, two reports have indicated how NTP may have a potential role here. Lin et al. provided the first in vivo report on NTP‐induced ICD in a colorectal cancer mouse model.^12^ In that study, NTP was combined with a therapeutic cancer vaccine, and tumors primed with NTP treatment demonstrated a higher specific T‐cell response. Furthermore, an indication of epitope spreading was also shown. Chen et al. was the first to combine ICD‐inducing NTP therapy with an anti‐PD‐L1 antibody.^54^ Not only did they report higher T‐cell infiltration into the tumor tissue, but the combination resulted in greater mouse survival. In the context of our study, these results are counterintuitive, as we demonstrated that PD‐L1 expression in the tumor decreased, which suggests less targets for the anti‐PD‐L1 therapy. To understand this seeming paradox, fundamental insight into the balance between the immunosuppressive impact of PD‐L1 and the threshold level of potential targets is required. Altogether, while studies combining NTP with immunotherapies have only just started, the adjuvant effects of NTP treatment appear promising and should be further investigated.

Finally, the practicality of NTP treatment of patients must also be considered as this technology moves toward clinical translation. Under research laboratory settings with stationary in vitro cancer models (e.g., monolayer cell cultures, 3D spheroids), treatment parameters are easily‐ and well‐controlled, but much of this control is lost when treatment is moved to the hospital setting due to practicality and treatment subject. Currently, NTP therapy relies heavily on clinician judgment and experience, as these devices are handheld and operated by the clinician.^26^, ^27^ Consequently, this leads to large variability which is further amplified when the treatment area is significantly larger than the NTP applicator and translation across the lesion is necessary. Furthermore, spatial displacement of the treatment area due to the breathing motion of the patient, a phenomenon referred to as respiratory‐induced tumor motion,^73^ can also affect off‐target treatment and NTP therapy response. This is also observed in our in vivo study, in which the respiration of the mouse can change the distance between the tumor and the DBD applicator. While the NTP energy per pulse is relatively the same when the application distance is between 1 and 4 mm, it is dramatically reduced at larger deviations (Figure 1h). Indeed, this is crucial as we have previously identified that NTP treatment energy may be an essential link to treatment response,^32^ and therefore, precise control over NTP therapy is necessary.

Some of these challenges are not exclusive to NTP and are also faced by radiation technology. Therefore, we can begin to address these challenges by incorporating techniques and experiences from the field of radiotherapy. In fact, our laboratory is looking to incorporate real‐time tumor tracking and robotic control with NTP therapy. This is a methodology used in radiotherapy that applies active compensatory motion of the radiation source with the tumor to counteract any tumor wandering.^74^, ^75^ By applying advances in tumor tracking technology and precision robotics, we may be able to address these challenges and reduce the variability of NTP therapy associated with both the operator and the patient.

## 4 CONCLUSION

It is clear that the field of “plasma oncology” has just started to scratch the surface into understanding how NTP affects not only the tumor but also the TME and the cancer‐immunity cycle at large. Here, we provided a detailed examination into how NTP treatment affects downstream, anti‐cancer immune responses over time in vivo. We also characterized the electrical and thermal properties of NTP therapy in situ over a range of application distances. These results showed that NTP was thermally and electrically well‐tolerated and provided insight into the stability of local NTP application. Studies into NTP characterization in more clinically‐relevant settings will further facilitate translation of this technology, and more in‐depth investigations into the holistic effects of NTP treatment is critically required. This will shed light on the potential niche for NTP treatment in oncotherapy via strategic combination with other treatment options.

## 5 MATERIALS AND METHODS

### 5.1 Cell line and culture

B16F10 melanoma cells were acquired from American Type Culture Collection (ATCC), expanded, and stored in liquid nitrogen. Cells were thawed 1 week before injection for in vivo studies and cultured in complete DMEM medium containing 10% fetal bovine serum, 100 U/ml penicillin, 100 μl streptomycin, and 4 × 10^−3^ M l‐glutamine. Cells were cultured at 37°C in a humidified environment with 5% CO_2_ and checked routinely for mycoplasma contamination. Cells used for subcutaneous injection were always between passage number 7–9, and cell counting was performed with a TC20 Automated Cell Counter (Bio‐Rad) after staining with 0.4% trypan blue.

### 5.2 In vivo melanoma model

Female, C57BL/6J, 8‐week‐old mice were purchased from Charles River and housed in a pathogen‐free room at the Animal Center of the University of Antwerp. Within each cage, two to three mice were randomly assigned to a treatment group (e.g., untreated control, NTP). The right flank of the mice was shaved 1 day before tumor inoculation. B16F10 melanoma cells were prepared in PBS and subcutaneously injected into the right flank of each mouse (10^6^ cells in 100 μl per mouse). Treatment was initiated 3 days later when tumors become palpable (~20 mm^3^). Tumor size and kinetics were followed up until the humane endpoints were reached. Two orthogonal diameters were measured using digital calipers, and volumes were calculated using 0.5 × length × width^2^. All animal experiments were approved by the University of Antwerp Animal Research Ethical Committee (ECD‐dossier 2017‐53).

### 5.3 NTP treatment

A microsecond‐pulsed dielectric barrier discharge (DBD) system previously described, was used for all NTP treatments.^33^ Briefly, a microsecond pulser (Megaimpulse Ltd., Russia) generated a 30 kV output pulse with rise time fixed within 1–1.5 μs and a pulse width of 2 μs. The frequency of the pulses was fixed at 700 Hz and treatment was performed for 5 consecutive days. The applicator of the system was a copper electrode, covered with a quartz dielectric, and was connected to the output of the microsecond pulser. The applicator was held by hand above the tumor (~1–3 mm) for treatment. Here, an electrically safe plasma was created in direct contact with the tumor, and the surrounding gas and tissue were not significantly heated.

### 5.4 Power measurements

To determine the plasma power during in vivo treatment of tumors, voltage and current was measured on the skin surface of a mouse during NTP treatment (Figure 1d). A mouse was sacrificed on the day of measurement and the hair was removed before treatment. The NTP applicator was fixed at a specified distance above the skin surface using a z‐positioner, and NTP was generated at 700 Hz. Voltage was measured using a 1000X high‐voltage probe (P6015A, Tektronix), and the current was measured with a current monitor (4100, Pearson Electronics, Inc.). The voltage and current waveforms were recorded on an oscilloscope (DSOX1102G, Keysight) with a 50 ns sampling rate. Voltage and current was used to determine instantaneous power (*P*(*t*)): *P*(*t*) = *V*(*t*) × *I*(*t*). The energy per pulse (*ε*_pulse_) was calculated: $\varepsilon_{\text{pulse}}=\sum_{k=1}^{n}\left|V_{k}\left(t\right)\times I_{k}\left(t\right)\right|\times ∆t$, where ∆*t* was 50 nanoseconds and the number of recorded samples (*n*) was 2000. In order to account for displacement current, the energy per pulse from displacement current (*ε*_pulse(displacement)_) was also measured when the high‐voltage pulse was applied to the NTP applicator 30 mm above the mouse. This value was subtracted from all other energy measurements. Taken together, the energy per pulse reported here (ε_pulse_) is: ε_pulse_ = ε_pulse(discharge)_‐ ε_pulse(displacement)_. The energy per pulse was measured over a range of application distances (1–10 mm).

### 5.5 Thermal imaging

The thermal images shown in Figure 1 are recorded using a cooled FLIR x6540sc thermal imaging camera. The camera has an InSb detector (2–5 μm) with a resolution of 640 × 512 pixels. It has a measurement accuracy of ±1°C and a thermal sensitivity/NETD (Noise Equivalent Temperature Difference) <25 mK. All NTP discharges were observed with a framerate of 30 fps and the image sequences were recorded using the FLIR Researcher IR Max software. Afterward, all plots were generated in Mathworks Matlab.

### 5.6 RNA sequencing and transcriptome analysis

For RNA sequencing, tumors were harvested on Days 7, 10, and 14 which correspond to immediately, 3 days, and 7 days after the last NTP treatment. Tissue was immediately weighed and transferred into RNAlater reagent (Qiagen). Total RNA was extracted and purified from excised mouse tumor tissue using the PureLink RNA Mini Kit (Invitrogen), according to the manufacturer's protocol. RNA concentration and purity was measured with an Epoch spectrophotometer (BioTek) by measuring absorbance at the 260/280 nm ratio. Sample were frozen in dry ice and shipped to BGI Genomics (BGI Group, China) for sequencing.

mRNA library preparation and sequencing was carried out by BGI Genomics with their proprietary BGISEQ‐500 platform and the oligo‐dt library. Briefly, mRNA was enriched and purified using oligo dT selection. RNA fragments were then reverse transcribed, ends repaired, adaptors ligated and amplified with PCR. Strands were separated, cyclized, and subjected to DNA nanoball synthesis. Libraries were 2 × 150 bp paired‐end sequenced on the DNBseq platform.

Reads were aligned to the Mus musculus GRCm38 (Ensembl, “top‐level” assembly) reference genome with STAR v2.7.2b using default alignment parameters.^76^ STAR was also used to generate gene counts tables (−‐quantMode GeneCounts). Normalization and differential expression analysis were carried out with DESeq2 v1.28.1. GSEA was performed using the hallmark gene sets from the public Molecular Signatures Database v7.4 (Broad Institute, USA). A preranked list was compiled based on a significance score that combines fold change and p value as described by Xiao et al.: $\pi_{i}=\log2\text{fold chang}e_{i}•\left(-\log_{10}p_{i}\right)$.^34^ An FDR ≤ 0.05 was set as the threshold for affected pathways.

### 5.7 Flow cytometry analysis

Characterization of tumor‐infiltration lymphocytes (TILs) was performed using multicolor flow cytometry experiments performed on mouse tumors, spleens and TDLN. At Days 7, 10, and 14, mice were sacrificed and tumors, spleens, and TDLN were removed and weighed. To obtain single‐cell suspensions, tumors were washed with FACS buffer and strained through a 70 μm cell strainer by mechanical dissociation. Likewise, TDLN and spleens were washed with FACS buffer and strained over a 40 μm cell strainer. Samples obtained from tumors and TDLN were stained with two different multicolor antibody panels (i.e., a T‐cell panel and DC/NK cell panel). Details concerning the flow cytometry panels and dilutions are described in supplementary information (Table S3). Prior to antibody staining, all single‐cell suspensions were pretreated using Fc blocking antibody (Clone 2.4G2, BD Biosciences) to avoid non‐specific binding to the Fc receptor and washed with FACS buffer. All samples were analyzed using the FACS Aria II flow cytometer (BD Biosciences).

### 5.8 Immunohistochemistry analysis

IHC stainings were performed on a Benchmark Ultra XT autostainer (Ventana Medical Systems Inc, Roche) according to the manufacturer's datasheet for anti‐CD4 (clone SP35, RTU, Ventana) and anti‐CD8 (clone SP57, RTU, Ventana). Slides for CD11c staining (clone N418, MA11C5, ThermoFisher) were performed manually. After staining, sections were counterstained with hematoxylin, washed, dehydrated, cleared in xylene, and mounted with Quick‐D Mounting Medium (Klinipath); then a cover slip was applied. Positive controls were included which consist of mice spleens. All slides were scored by one pathologist and one scientist based on location (periphery or tumor center) and density (0 = empty, 1 = sparse, 2 = moderate, 3 = dense) using an Olympus BX41 microscope. Pictures were taken using the Leica acquisition software version 4.

### 5.9 Immunofluorescence analysis

Tumor, resected on Days 7, 10, and 14, were immediately fixed in 4% paraformaldehyde overnight at room temperature, dehydrated and paraffin embedded the next day, and sectioned at 5 μm per slice. Tumor sections were stored at 4°C until the day of staining, where they were deparaffinized and rehydrated. Heat‐induced antigen retrieval with sodium citrate buffer (10 mM sodium citrate, 0.05% Tween 20, pH 6.0) was performed at 96°C for 20 mins. Slides were washed twice for 5 min with a wash buffer (TBS + 0.024% Triton X‐100) before the blocking step. Tissue was blocked with 10% goat serum in the wash buffer for 2 h at room temperature. Incubation with the primary antibody was performed overnight at 4°C for CRT (1/500 dilution; clone 3EPR3924, Abcam, ab92516), CD47 (1/100 dilution; clone B6H12.2, ThermoFisher Scientific, MA5‐11895), and PD‐L1 (1/200 dilution; clone 10F.9G2, Biolegend, 124301). Dilutions were optimized by comparing each primary stain with the corresponding isotype staining of the same concentrations: Rabbit IgG isotype control (ThermoFisher Scientific, 10500C) for CRT, Rat IgG2a kappa isotype control (ThermoFisher Scientific, 14‐4321‐82) for CD47, and Rat IgG2b kappa isotype control (Biolgend, 400601) for PD‐L1. Comparison of primary staining with isotype controls are provided in supporting information (Figure S7). All slides were washed with wash buffer (two times for 5 min) and stained with the secondary antibody for 1 h in the dark at room temperature: Goat anti‐rabbit IgG (H + L) AlexaFluor488 (1/1000 dilution; Abcam, ab150077) for CRT and Goat anti‐rat IgG (H + L) AlexaFluor594 (1/200 dilution; ThermoFisher Scientific, A‐11007) for CD47 and PD‐L1. All slides were stained with Sudanschwarz B (0.1% in 70% ethanol; Merck, CI26150) for 30 min to reduce background staining. Lastly, all slides were washed three times for 5 min with the wash buffer, mounted with VECTASHIELD® Antifade mounting medium containing counter staining DAPI (H‐1200, VECTOR laboratories), and stored in the dark at 4°C until imaging. All slides from tissue collected on the same day were imaged together at the same capture parameters with the Olympus BX51 fluorescence microscope (Olympus Life Sciences, Cat. No. WS‐BX51‐0169) in order to reduce variability.

For Treg identification, slides were dual stained with CD3 (1/200; clone 4SM95, ThermoFisher, 14‐9766‐82) and FOXP3 (1/500; clone FJK‐16s, ThermoFisher Scientific, 14‐5773‐82). Staining concentrations were determined based on corresponding isotype controls, Rat IgG1, kappa (Biolegend, 400401) and Rat IgG2a, kappa (Biolegend, 400501), respectively. Goat anti‐rabbit IgG (H + L) AlexaFluor488 and Goat anti‐rat IgG (H + L) AlexaFluor594 secondary antibodies were also used at 1/500 dilutions. All tumor slides were imaged on the same day, as detailed above, and batch processed with our software in order to reduce variability.

### 5.10 Computational image processing

Fluorescent microscopy images were taken at 10x with the Olympus BX51 fluorescence microscope (Olympus Life Sciences, Cat. No. WS‐BX51‐0169) were measured by using raw fluorescent DAPI to create a binary mask for the locations of the individual nuclei. This was accomplished by removing background signals and artifacts in the DAPI images via the use of a one‐sided low‐pass filter formed by a Gaussian kernel of 40 pixels in standard deviation. Resultant values were saturated to 95th percentile of the image intensities and binarized to 0.40 of the normalized dynamic range as described in our previous work.^33^ These details are further described in the supplementary information.

To quantify Treg counts in the tumor section, individual cell positivity was measured by taking the background‐subtracted intensity values and binarizing the intensity values using a threshold of 0.2 for TxRed (FOXP3) and 0.3 for GFP(CD3). Masked pixels that did not overlap with either nuclei or cytoplasm were removed. Similar to the analysis for DAPI, binary objects were indexed using connected component analysis and small objects (less than 20 pixels) were filtered. In this case, large objects greater than 500 pixels were also filtered. Cells that contained fluorescent signal overlapping with either the nuclei or the cytoplasm were counted and those that contained both fluorescent markers were considered to be double positive.

### 5.11 Statistical analysis

Statistical differences for this study were analyzed using the linear mixed model with JMP Pro 13 (SAS software), where NTP treatment was set as the fixed effect. The fixed effect test was used to determine statistical differences (*p* ≤ 0.05), and the student's t‐test was used to determine the *p* value when comparing NTP treatment to untreated controls, post hoc. Data are represented as mean ± SEM and all individual values are reported. For survival analysis of the in vivo studies, the log‐rank (Mantel‐Cox) test was used and a *p* value ≤ 0.05 was considered statistically significant. Two independent experiments were performed. All graphs were made in Graphpad Prism 8 (Graphpad Software).

## AUTHOR CONTRIBUTIONS

**Abraham Lin:** Conceptualization (lead); data curation (lead); formal analysis (lead); funding acquisition (equal); investigation (lead); methodology (lead); project administration (equal); validation (lead); visualization (lead); writing – original draft (lead); writing – review and editing (lead). **Joey De Backer:** Data curation (supporting); investigation (supporting); methodology (supporting); writing – original draft (supporting); writing – review and editing (supporting). **Delphine Quatannens:** Data curation (supporting); formal analysis (supporting); funding acquisition (equal); investigation (supporting); methodology (supporting); writing – original draft (supporting); writing – review and editing (supporting). **Bart Cuypers:** Data curation (supporting); investigation (supporting); methodology (supporting); validation (supporting); visualization (supporting); writing – original draft (supporting); writing – review and editing (supporting). **Hanne Verswyvel:** Funding acquisition (equal); investigation (supporting); methodology (supporting); writing – original draft (supporting); writing – review and editing (supporting). **Edgar Cardenas De La Hoz:** Investigation (supporting); methodology (supporting); software (lead); validation (supporting); writing – original draft (supporting); writing – review and editing (supporting). **Bart Ribbens:** Formal analysis (supporting); investigation (supporting); methodology (supporting); visualization (supporting); writing – original draft (supporting); writing – review and editing (supporting). **Vasiliki Siozopoulou:** Formal analysis (supporting); investigation (supporting); validation (supporting); visualization (supporting); writing – original draft (supporting); writing – review and editing (supporting). **Jonas Van Audenaerde:** Investigation (supporting); methodology (supporting); writing – original draft (supporting); writing – review and editing (supporting). **Elly Marcq:** Methodology (supporting); writing – original draft (supporting); writing – review and editing (supporting). **Filip Lardon:** Project administration (equal); resources (equal); writing – original draft (supporting); writing – review and editing (supporting). **Kris Laukens:** Project administration (supporting); resources (equal); supervision (equal); writing – original draft (supporting); writing – review and editing (supporting). **Steve Vanlanduit:** Project administration (supporting); resources (equal); supervision (equal); writing – original draft (supporting); writing – review and editing (supporting). **Evelien Smits:** Funding acquisition (supporting); project administration (supporting); resources (equal); supervision (equal); writing – original draft (supporting); writing – review and editing (supporting). **Annemie Bogaerts:** Funding acquisition (equal); project administration (equal); resources (equal); supervision (equal); writing – original draft (supporting); writing – review and editing (supporting).

## Supporting information


Appendix S1 Supporting Information

**Video S1** XXX

**Video S2** XXX

## Data Availability

The data that support the findings of this study are available from the corresponding author upon reasonable request.
