# Supplementary material for: The effect of local non‐thermal plasma therapy on the cancer‐immunity cycle in a melanoma mouse model
Source: Bioeng Transl Med. 2022 Apr 21;7(3):e10314. doi: 10.1002/btm2.10314 (PMC9472020; doi:10.1002/btm2.10314)
Supplement: Supplementary file 1 — Appendix S1 Supporting Information [file BTM2-7-e10314-s001.docx]

Supporting Information

The Effect of Local Non-Thermal Plasma Therapy on the Cancer-Immunity Cycle in a Melanoma Mouse Model

*Abraham Lin^1,2^*, Joey De Backer^3^, Delphine Quatannens^2^, Bart Cuypers^4^, Hanne Verswyvel^1,2^, Edgar Cardenas De La Hoz^5^, Bart Ribbens^5^, Vasiliki Siozopoulou^6^, Jonas Van Audenaerde^2^, Elly Marcq^2^, Filip Lardon^2^, Kris Laukens^4^, Steve Vanlanduit^5^, Evelien Smits^2^, Annemie Bogaerts^1^*

1. Optimizing NTP treatment intensity for melanoma treatment

In order to determine the optimal NTP treatment parameters, subcutaneous B16F10 melanoma tumors were treated with NTP for 10 seconds at different intensities (defined by the pulse frequency: 500, 700, and 1000 Hz) for 5 consecutive days (n=4-5) and monitored up to day 17 in a small pilot study (**Figure S1**). Compared to the untreated controls (541.6±256.8 mm^3^), 700 Hz treatment had the greatest effect on reducing tumor volumes (230.6±96.4 mm^3^) on day 17. At a lower treatment intensity (500 Hz), treatment did not affect tumor volume (419.1±127.7 mm^3^) and further increase in pulse frequency to 1000 Hz did not benefit therapy response (272.2±131.4 mm^3^). Therefore, NTP treatment intensity of 700 Hz was considered the most optimal treatment and was used in all subsequent experiments.


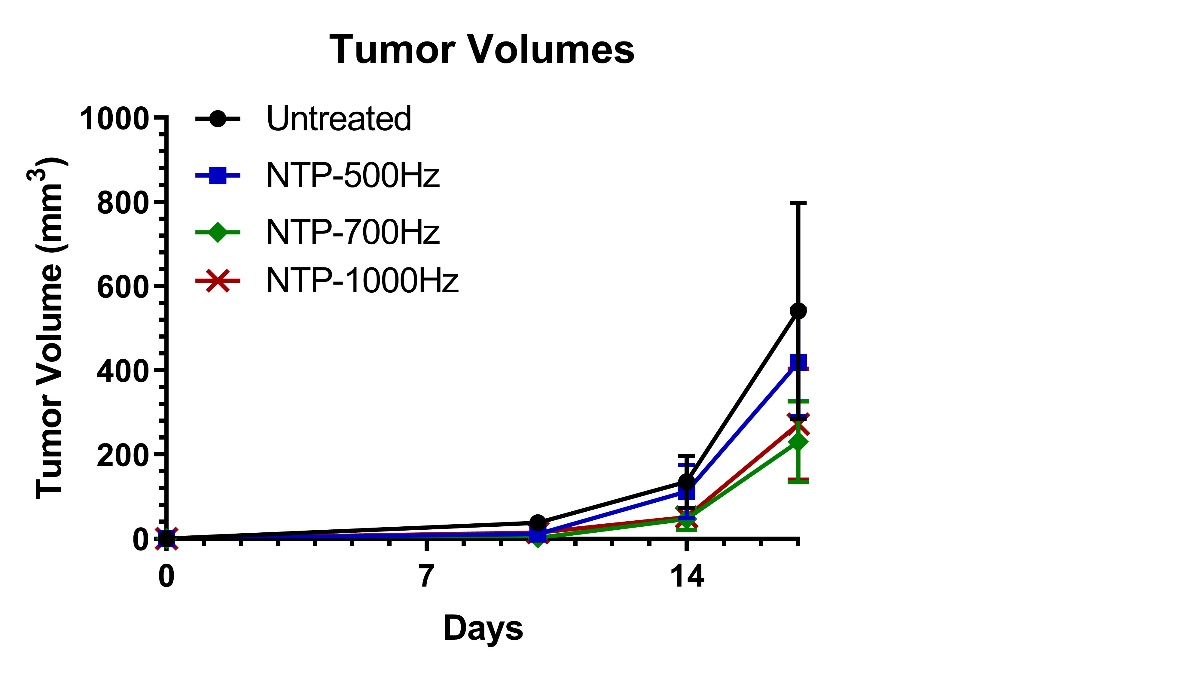


Figure S1. Assessment of the anti-cancer NTP effects at different treatment intensities (defined by the pulse frequency). Subcutaneous melanoma tumors were treated for 5 consecutive days with NTP and monitored up to day 17 (n=4-5).

2. Thermography analysis

## 2.1 Temperature evolution of NTP treatment for 10 seconds

A video showing the temperature evolution of NTP treatment on the mouse skin, as well as the cooling profile is shown in **Supplementary Video 1**. Images taken immediately after NTP treatment also indicate that no visible damage had occurred during treatment, thus further suggesting that the thermal properties of NTP are not associated with its therapeutic effect (**Figure S2**).


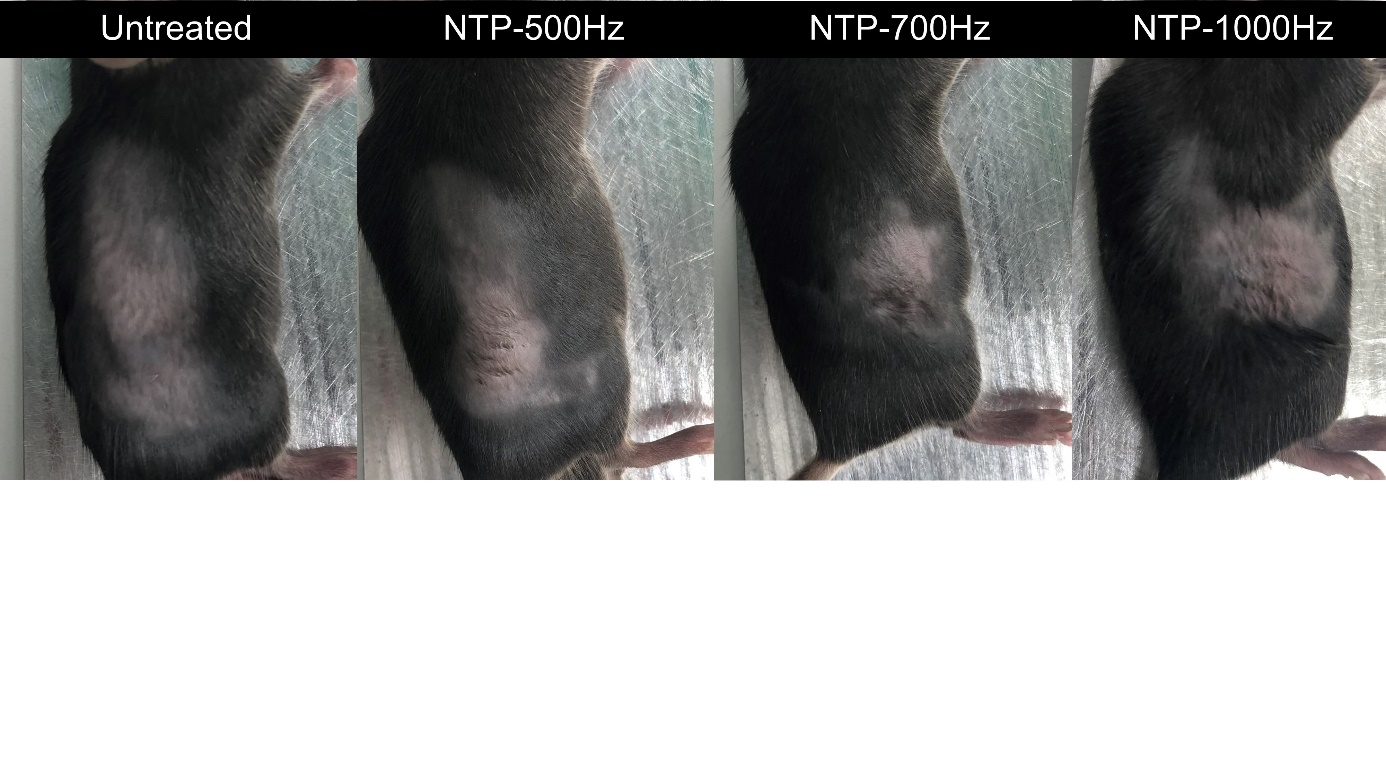


Figure S2. Images of the mouse skin taken after NTP treatment at various treatment intensities (defined by pulse frequency). The hair was removed and NTP was discharged directly onto the skin of the mouse. No visible or thermal damage was observed following treatment compared to untreated.

## 2.2 Temperature evolution of NTP treatment for 60 seconds

The effect of NTP treatment over longer application times was also investigated. The temperature of the mouse skin did not increase past 38^o^C with 60 seconds of NTP treatment and rapidly cooled to baseline when treatment was stopped (**Figure S3a**). A spatial profile from the point directly below the NTP applicator (**Figure S3b**) showed that the temperature of the skin 9 mm from the center of treatment was unaffected (**Figure S3c**). A video showing this temperature evolution is shown in **Supplementary Video 2**.


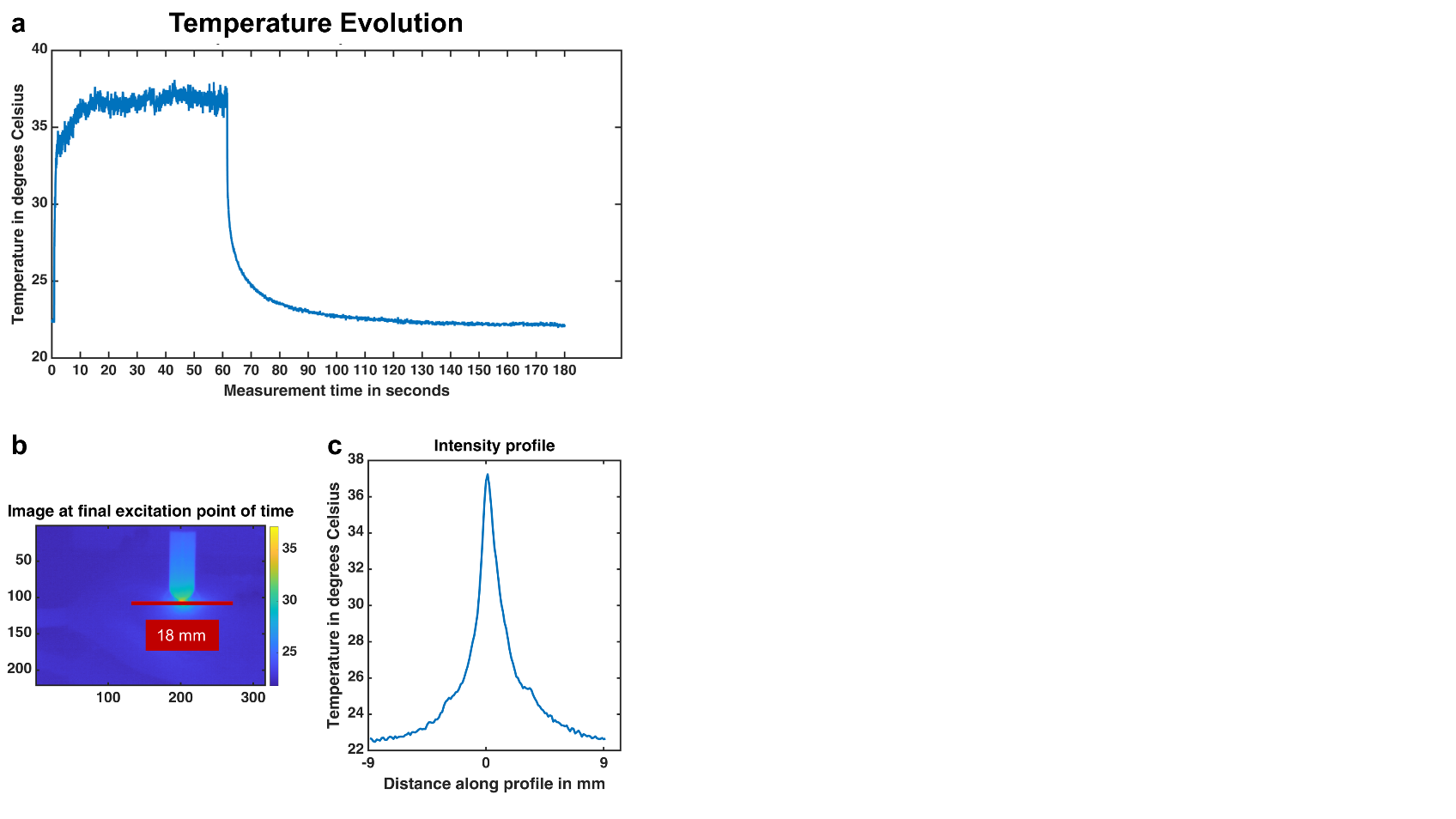


Figure S3. Thermal effects of extended NTP treatment on mouse skin. a) NTP treatment of 60 seconds showed that temperature of the skin below the NTP applicator does not increase past 38^o^C and cools rapidly when treatment was stopped. b) The temperature of the skin was measured immediately after NTP treatment and c) the spatial profile showed that the temperature of skin 9 mm from the center of treatment was unaffected.

3. RNA sequencing analysis

## 3.1 GSEA Analysis

A pre-ranked GSEA analysis was performed on RNA sequences from tumors resected on day 7, 10, and 14, comparing that of NTP-treated to untreated controls. A complete list of the upregulated and downregulated hallmark gene sets (adjusted p-value ≤ 0.05) is provided (**Table S1**).

Table S1. Upregulated and downregulated hallmark gene sets

| **Day 7** | | | | | | | |
| --- | --- | --- | --- | --- | --- | --- | --- |
| *NAME* | *SIZE* | *NES* | *NOM*  *p-val* | *FDR*  *q-val* | *FWER*  *p-val* | *RANK AT MAX* | *LEADING EDGE* |
| HALLMARK_CHOLESTEROL_HOMEOSTASIS | 64 | 1.419 | 0.028 | 0.518 | 0.288 | 3856 | tags=55%, list=26%, signal=73% |
| HALLMARK_E2F_TARGETS | 200 | 1.369 | 0.004 | 0.404 | 0.411 | 6032 | tags=63%, list=40%, signal=103% |
| HALLMARK_UNFOLDED_PROTEIN_RESPONSE | 110 | 1.300 | 0.046 | 0.476 | 0.616 | 4495 | tags=45%, list=30%, signal=64% |
| HALLMARK_MYC_TARGETS_V1 | 200 | 1.282 | 0.031 | 0.418 | 0.673 | 6708 | tags=69%, list=45%, signal=122% |
| HALLMARK_INTERFERON_GAMMA_RESPONSE | 165 | -2.178 | 0.000 | 0.000 | 0.000 | 2512 | tags=70%, list=17%, signal=83% |
| HALLMARK_INTERFERON_ALPHA_RESPONSE | 85 | -2.078 | 0.000 | 0.000 | 0.000 | 1875 | tags=73%, list=12%, signal=83% |
| HALLMARK_MYOGENESIS | 143 | -2.022 | 0.000 | 0.000 | 0.000 | 1905 | tags=31%, list=13%, signal=36% |
| HALLMARK_ALLOGRAFT_REJECTION | 145 | -1.807 | 0.000 | 0.000 | 0.001 | 1842 | tags=49%, list=12%, signal=55% |
| HALLMARK_IL6_JAK_STAT3_SIGNALING | 56 | -1.647 | 0.008 | 0.003 | 0.023 | 3165 | tags=54%, list=21%, signal=68% |
| HALLMARK_IL2_STAT5_SIGNALING | 156 | -1.614 | 0.000 | 0.005 | 0.043 | 2981 | tags=33%, list=20%, signal=41% |
| HALLMARK_INFLAMMATORY_RESPONSE | 131 | -1.499 | 0.002 | 0.022 | 0.190 | 2144 | tags=36%, list=14%, signal=41% |
| HALLMARK_COMPLEMENT | 144 | -1.429 | 0.016 | 0.049 | 0.424 | 3211 | tags=42%, list=21%, signal=52% |
| **Day 10** | | | | | | | |
| *NAME* | *SIZE* | *NES* | *NOM*  *p-val* | *FDR*  *q-val* | *FWER*  *p-val* | *RANK AT MAX* | *LEADING EDGE* |
| HALLMARK_OXIDATIVE_PHOSPHORYLATION | 137 | 2.034 | 0.000 | 0.000 | 0.000 | 939 | tags=52%, list=18%, signal=62% |
| HALLMARK_HYPOXIA | 103 | 2.012 | 0.000 | 0.000 | 0.000 | 393 | tags=35%, list=8%, signal=37% |
| HALLMARK_MTORC1_SIGNALING | 154 | 2.011 | 0.000 | 0.000 | 0.000 | 634 | tags=26%, list=12%, signal=29% |
| HALLMARK_FATTY_ACID_METABOLISM | 76 | 1.934 | 0.000 | 0.000 | 0.000 | 743 | tags=34%, list=14%, signal=39% |
| HALLMARK_GLYCOLYSIS | 107 | 1.910 | 0.000 | 0.000 | 0.000 | 592 | tags=31%, list=11%, signal=34% |
| HALLMARK_MYC_TARGETS_V1 | 177 | 1.777 | 0.000 | 0.001 | 0.006 | 1252 | tags=46%, list=24%, signal=58% |
| HALLMARK_HEME_METABOLISM | 85 | 1.711 | 0.003 | 0.004 | 0.025 | 533 | tags=26%, list=10%, signal=28% |
| HALLMARK_INTERFERON_ALPHA_RESPONSE | 38 | 1.703 | 0.003 | 0.004 | 0.028 | 682 | tags=39%, list=13%, signal=45% |
| HALLMARK_XENOBIOTIC_METABOLISM | 65 | 1.671 | 0.002 | 0.005 | 0.045 | 857 | tags=28%, list=16%, signal=33% |
| HALLMARK_P53_PATHWAY | 84 | 1.656 | 0.000 | 0.006 | 0.055 | 971 | tags=32%, list=19%, signal=39% |
| HALLMARK_INTERFERON_GAMMA_RESPONSE | 77 | 1.625 | 0.000 | 0.008 | 0.086 | 682 | tags=29%, list=13%, signal=32% |
| HALLMARK_PI3K_AKT_MTOR_SIGNALING | 64 | 1.599 | 0.008 | 0.010 | 0.111 | 355 | tags=17%, list=7%, signal=18% |
| HALLMARK_UNFOLDED_PROTEIN_RESPONSE | 84 | 1.479 | 0.010 | 0.040 | 0.399 | 850 | tags=23%, list=16%, signal=27% |
| HALLMARK_ADIPOGENESIS | 115 | 1.477 | 0.016 | 0.037 | 0.401 | 697 | tags=23%, list=13%, signal=26% |
| HALLMARK_UV_RESPONSE_UP | 83 | 1.356 | 0.045 | 0.099 | 0.795 | 666 | tags=20%, list=13%, signal=23% |
| HALLMARK_EPITHELIAL_MESENCHYMAL_TRANSITION | 89 | -2.139 | 0.000 | 0.000 | 0.000 | 427 | tags=39%, list=8%, signal=42% |
| HALLMARK_COAGULATION | 43 | -1.754 | 0.003 | 0.007 | 0.016 | 381 | tags=26%, list=7%, signal=27% |
| HALLMARK_MYOGENESIS | 65 | -1.716 | 0.000 | 0.011 | 0.033 | 551 | tags=26%, list=11%, signal=29% |
| HALLMARK_UV_RESPONSE_DN | 78 | -1.669 | 0.005 | 0.014 | 0.059 | 575 | tags=27%, list=11%, signal=30% |
| HALLMARK_MITOTIC_SPINDLE | 143 | -1.355 | 0.027 | 0.226 | 0.699 | 711 | tags=23%, list=14%, signal=26% |
| **Day 14** | | | | | | | |
| *NAME* | *SIZE* | *NES* | *NOM p-val* | *FDR*  *q-val* | *FWER*  *p-val* | *RANK AT MAX* | *LEADING EDGE* |
| HALLMARK_MYC_TARGETS_V1 | 200 | 1.874 | 0.000 | 0.002 | 0.002 | 1307 | tags=40%, list=11%, signal=44% |
| HALLMARK_E2F_TARGETS | 199 | 1.750 | 0.000 | 0.031 | 0.034 | 1332 | tags=35%, list=11%, signal=38% |
| HALLMARK_MYC_TARGETS_V2 | 57 | 1.589 | 0.046 | 0.119 | 0.182 | 2147 | tags=63%, list=18%, signal=77% |
| HALLMARK_FATTY_ACID_METABOLISM | 128 | 1.508 | 0.006 | 0.155 | 0.284 | 409 | tags=5%, list=4%, signal=5% |
| HALLMARK_PEROXISOME | 79 | 1.464 | 0.074 | 0.169 | 0.358 | 913 | tags=14%, list=8%, signal=15% |
| HALLMARK_G2M_CHECKPOINT | 195 | 1.416 | 0.017 | 0.186 | 0.456 | 1362 | tags=30%, list=12%, signal=34% |
| HALLMARK_MTORC1_SIGNALING | 198 | 1.344 | 0.023 | 0.175 | 0.591 | 1261 | tags=24%, list=11%, signal=27% |
| HALLMARK_INTERFERON_GAMMA_RESPONSE | 175 | -1.785 | 0.000 | 0.000 | 0.000 | 557 | tags=53%, list=5%, signal=54% |
| HALLMARK_INTERFERON_ALPHA_RESPONSE | 90 | -1.762 | 0.000 | 0.000 | 0.000 | 463 | tags=69%, list=4%, signal=71% |
| HALLMARK_IL6_JAK_STAT3_SIGNALING | 68 | -1.627 | 0.000 | 0.003 | 0.010 | 542 | tags=22%, list=5%, signal=23% |
| HALLMARK_INFLAMMATORY_RESPONSE | 144 | -1.621 | 0.000 | 0.003 | 0.013 | 834 | tags=28%, list=7%, signal=30% |
| HALLMARK_COMPLEMENT | 144 | -1.607 | 0.001 | 0.003 | 0.020 | 993 | tags=24%, list=9%, signal=26% |
| HALLMARK_ALLOGRAFT_REJECTION | 148 | -1.552 | 0.001 | 0.010 | 0.070 | 807 | tags=30%, list=7%, signal=32% |
| HALLMARK_APOPTOSIS | 138 | -1.529 | 0.001 | 0.015 | 0.116 | 1319 | tags=25%, list=11%, signal=27% |
| HALLMARK_COAGULATION | 90 | -1.502 | 0.009 | 0.024 | 0.203 | 727 | tags=13%, list=6%, signal=14% |
| HALLMARK_UV_RESPONSE_DN | 132 | -1.453 | 0.007 | 0.049 | 0.407 | 610 | tags=7%, list=5%, signal=7% |
| HALLMARK_TNFA_SIGNALING_VIA_NFKB | 174 | -1.428 | 0.008 | 0.062 | 0.519 | 1225 | tags=22%, list=11%, signal=25% |
| HALLMARK_IL2_STAT5_SIGNALING | 163 | -1.386 | 0.015 | 0.095 | 0.720 | 1089 | tags=26%, list=9%, signal=28% |
| HALLMARK_EPITHELIAL_MESENCHYMAL_TRANSITION | 168 | -1.364 | 0.019 | 0.114 | 0.819 | 833 | tags=8%, list=7%, signal=9% |
| NES: normalized enrichment score; NOM p-val: adjusted p-value; FDR q-val: false discovery rate; FWER p-val: family-wise error rate | | | | | | | |

## 3.2 Differential Gene Expression (DESeq2) Analysis

Based on the GSEA analysis, we further investigated the genes in the IL6-JAK-STAT3, unfolded protein response, and interferon gamma pathways that were significantly (p≤0.05) up- or downregulated on day 7, 10, and 14 (**Table S2**). Due to the high variability within the mice, the adjusted p-values did not reveal any significant genes, and therefore, the uncorrected p-values and log2fold change values (Log2FC) are provided. These results should, therefore, be interpreted with care, and increasing the number of mice per group would help provide more concrete insight.

Table S2. Genes in the selected pathways that were upregulated or downregulated

| IL6-JAK-STAT3 | | | | | | | | |
| --- | --- | --- | --- | --- | --- | --- | --- | --- |
| Day 7 | | | **Day 10** | | | **Day 14** | | |
| *Gene* | *Log2FC* | *p-value* | *Gene* | *Log2FC* | *p-value* | *Gene* | *Log2FC* | *p-value* |
| Il12rb1 | -1.832 | 0.0705 | Il6st | -0.2571 | 0.0045 | Itgb3 | -0.6963 | 4.85E-08 |
| Stat1 | -0.882 | 0.1805 | Cbl | -0.2151 | 0.0197 | Tyk2 | -0.2327 | 0.0017 |
| Tnf | -0.830 | 0.1916 | Itga4 | -0.2697 | 0.0354 | Il12rb1 | -2.5049 | 0.0021 |
| Stat2 | -0.944 | 0.1932 | Osmr | -0.6945 | 0.1022 | Tnf | -1.1278 | 0.0210 |
| Irf1 | -0.925 | 0.2319 | Csf2ra | -0.5589 | 0.1128 | Stat2 | -1.1150 | 0.0233 |
| Cxcl9 | -0.955 | 0.2418 | Il1r1 | -1.1327 | 0.1174 | Jun | -0.3905 | 0.0371 |
| Il2rg | -1.028 | 0.2997 | Pf4 | -0.8373 | 0.1627 | Cxcl9 | -2.4841 | 0.0395 |
| Socs1 | -0.659 | 0.4365 | Cd14 | -0.9670 | 0.1649 | Stat1 | -1.2806 | 0.0423 |
| Cxcl10 | -0.684 | 0.4385 | Tlr2 | -0.6513 | 0.1667 | Irf1 | -1.7070 | 0.0447 |
| Il2ra | -0.605 | 0.4892 | Il12rb1 | 0.6042 | 0.1752 | Cntfr | 0.2303 | 0.0457 |
|  |  |  | Ccl7 | -0.6657 | 0.2857 | Irf9 | -0.7631 | 0.0603 |
|  |  |  | Pik3r5 | -0.5287 | 0.3935 | Il15ra | -0.8677 | 0.0818 |
|  |  |  | Csf2rb | -0.5583 | 0.3985 | Socs1 | -1.3700 | 0.0954 |
|  |  |  |  |  |  | Map3k8 | -0.5375 | 0.1362 |
|  |  |  |  |  |  | Il2ra | -1.4244 | 0.1562 |
|  |  |  |  |  |  | Cxcl10 | -0.8965 | 0.2094 |
|  |  |  |  |  |  | Il2rg | -0.5721 | 0.3507 |
|  |  |  |  |  |  | Csf2rb | -0.5208 | 0.3878 |
|  |  |  |  |  |  | Csf3r | -0.5685 | 0.4141 |
| Unfolded Protein Response | | | | | | | | |
| Day 7 | | | **Day 10** | | | **Day 14** | | |
| *Gene* | *Log2FC* | *p-value* | *Gene* | *Log2FC* | *p-value* | *Gene* | *Log2FC* | *p-value* |
| Vegfa | 0.6583 | 0.0191 | Gosr2 | 0.2464 | 0.0008 | Spcs3 | 0.2125 | 0.0076 |
| Ero1a | 0.5655 | 0.0481 | Eif4ebp1 | 0.2498 | 0.0039 | Ssr1 | 0.2370 | 0.0099 |
|  |  |  | Ero1a | 0.4929 | 0.0047 | Calr | 0.2265 | 0.0321 |
|  |  |  | Ddit4 | 0.4062 | 0.0054 | Exosc2 | 0.1815 | 0.0453 |
|  |  |  | Eif4a3 | 0.1968 | 0.0180 |  |  |  |
|  |  |  | Hspa9 | 0.2128 | 0.0297 |  |  |  |
|  |  |  | H2ax | 0.2244 | 0.0329 |  |  |  |
|  |  |  | Cxxc1 | -0.1469 | 0.0407 |  |  |  |
|  |  |  | Eif4a1 | 0.1121 | 0.0433 |  |  |  |
|  |  |  | Nop56 | -0.1647 | 0.0488 |  |  |  |
|  |  |  | Atp6v0d1 | 0.1725 | 0.0488 |  |  |  |
|  |  |  | Sec11a | 0.2217 | 0.0491 |  |  |  |
| Interferon Gamma | | | | | | | | |
| Day 7 | | | **Day 10** | | | **Day 14** | | |
| *Gene* | *Log2FC* | *p-value* | *Gene* | *Log2FC* | *p-value* | *Gene* | *Log2FC* | *p-value* |
| Nlrc5 | -1.1418 | 0.0622 | Ifi27 | 0.4000 | 0.0059 | Helz2 | -0.8278 | 0.0006 |
| Rnf213 | -0.8380 | 0.1077 | Pfkp | 0.3182 | 0.0070 | Znfx1 | -0.4797 | 0.0036 |
| Tap1 | -1.1387 | 0.1389 | Ube2l6 | 0.8084 | 0.0087 | Adar | -0.5821 | 0.0048 |
| Bst2 | -0.8724 | 0.1424 | Nampt | 0.3264 | 0.0108 | Samd9l | -0.8436 | 0.0086 |
| Gbp6 | -1.4343 | 0.1435 | Bst2 | 0.4497 | 0.0133 | Ifi27 | -0.5449 | 0.0115 |
| B2m | -0.8436 | 0.1516 | Ncoa3 | -0.2523 | 0.0211 | Ddx58 | -1.0325 | 0.0133 |
| Tapbp | -0.9466 | 0.1731 | Psmb2 | 0.2188 | 0.0383 | Pml | -0.3744 | 0.0169 |
| Psme1 | -0.6909 | 0.1782 | Plscr1 | 0.2033 | 0.0440 | Stat2 | -1.1150 | 0.0233 |
| Lgals3bp | -0.6387 | 0.1850 | Psma2 | 0.1782 | 0.0466 | Rsad2 | -1.2412 | 0.0238 |
| Stat2 | -0.9442 | 0.1932 | Tap1 | 0.5926 | 0.1588 | Ifitm2 | 0.2086 | 0.0245 |
| Ifitm3 | -0.8949 | 0.2090 | Ly6e | -0.5632 | 0.3677 | Trim25 | -0.7181 | 0.0279 |
| Parp12 | -0.7404 | 0.2141 | Gbp6 | 0.5554 | 0.4007 | Parp14 | -1.6582 | 0.0293 |
| Parp14 | -0.9491 | 0.2176 | Serping1 | -0.6143 | 0.4154 | Gbp6 | -2.6723 | 0.0302 |
| Ube2l6 | -0.9185 | 0.2263 |  |  |  | Ogfr | -0.3453 | 0.0327 |
| Znfx1 | -0.5331 | 0.2269 |  |  |  | Tapbp | -1.3173 | 0.0331 |
| Cxcl9 | -0.9551 | 0.2418 |  |  |  | Parp12 | -1.1176 | 0.0343 |
| Samhd1 | -0.9275 | 0.2427 |  |  |  | Nlrc5 | -1.9799 | 0.0392 |
| Cd74 | -1.1967 | 0.2444 |  |  |  | Cxcl9 | -2.4841 | 0.0395 |
| Samd9l | -0.6074 | 0.2455 |  |  |  | Lap3 | -0.4080 | 0.0461 |
| Lap3 | -0.5951 | 0.2580 |  |  |  | Ube2l6 | -1.3170 | 0.0470 |
| Irf2 | -0.5628 | 0.2593 |  |  |  | Ifitm3 | -0.8906 | 0.0486 |
| Rsad2 | -0.5874 | 0.4086 |  |  |  | Irf2 | -0.3023 | 0.0488 |
| Cxcl10 | -0.6842 | 0.4385 |  |  |  | Rnf213 | -0.9596 | 0.0532 |
|  |  |  |  |  |  | Tap1 | -1.8648 | 0.0568 |
|  |  |  |  |  |  | Irf9 | -0.7631 | 0.0603 |
|  |  |  |  |  |  | B2m | -1.0038 | 0.0649 |
|  |  |  |  |  |  | Psme1 | -0.8148 | 0.0740 |
|  |  |  |  |  |  | Lgals3bp | -0.5764 | 0.0861 |
|  |  |  |  |  |  | Bst2 | -0.9201 | 0.1174 |
|  |  |  |  |  |  | Cd74 | -1.2132 | 0.1771 |
|  |  |  |  |  |  | Cxcl10 | -0.8965 | 0.2094 |
|  |  |  |  |  |  | Samhd1 | -0.5023 | 0.2339 |
|  |  |  |  |  |  | Serping1 | -0.5226 | 0.4818 |

4. Flow cytometry analysis

## 4.1 DC and NK cell gating strategy

A representative gating strategy to identify dendritic cells (DCs) and natural killer (NK) cells is shown (**Figure S4**).


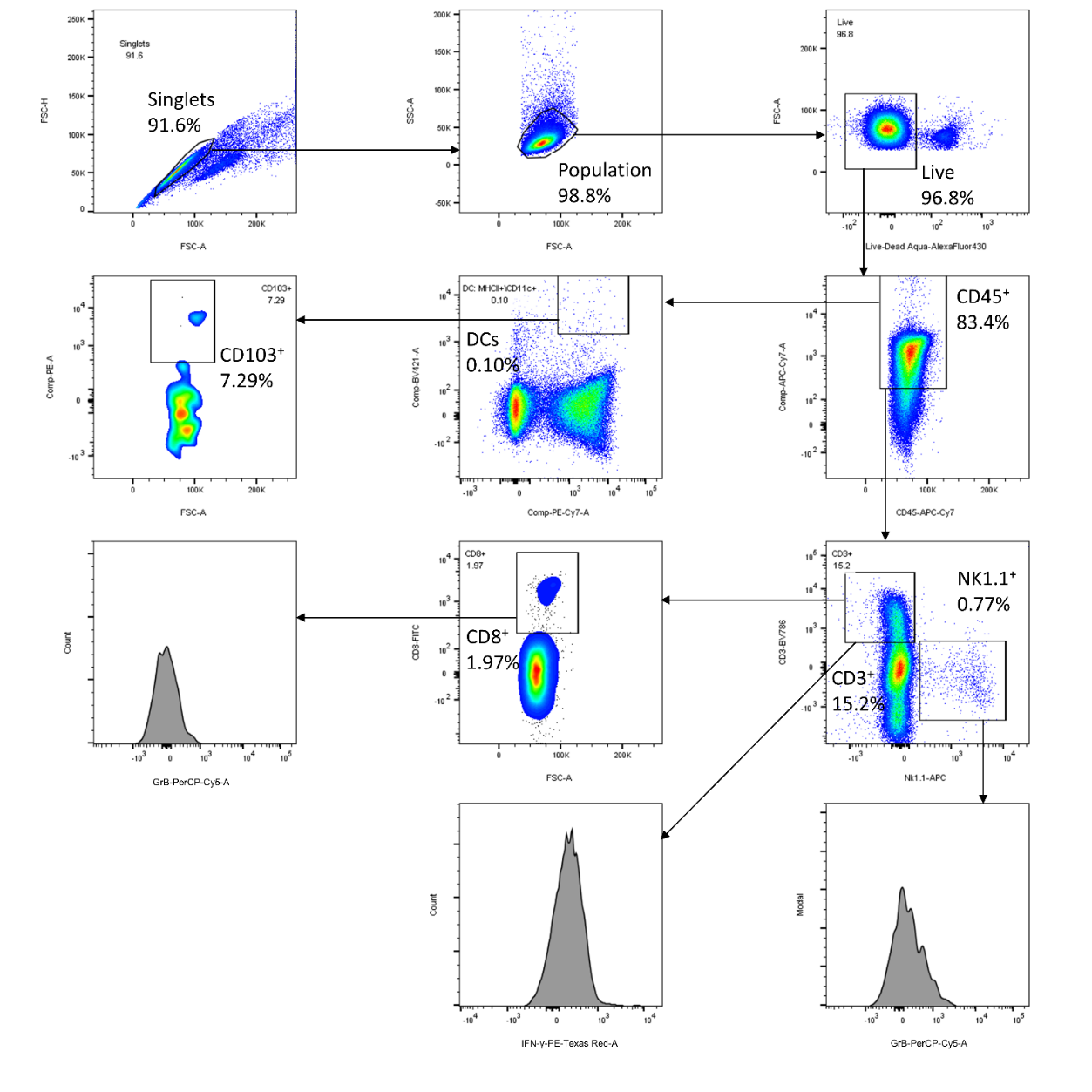


Figure S4. The flow cytometry gating strategy to identify DCs and NK cells along with intracellular interferon-gamma and granzyme b.

## 4.2 T cell gating strategy

A representative gating strategy to identify CD8^+^, non-regulatory CD4^+^, and regulatory T cells is shown (**Figure S5**). The gating strategy to delineate non-exhausted populations of T cells are also shown.


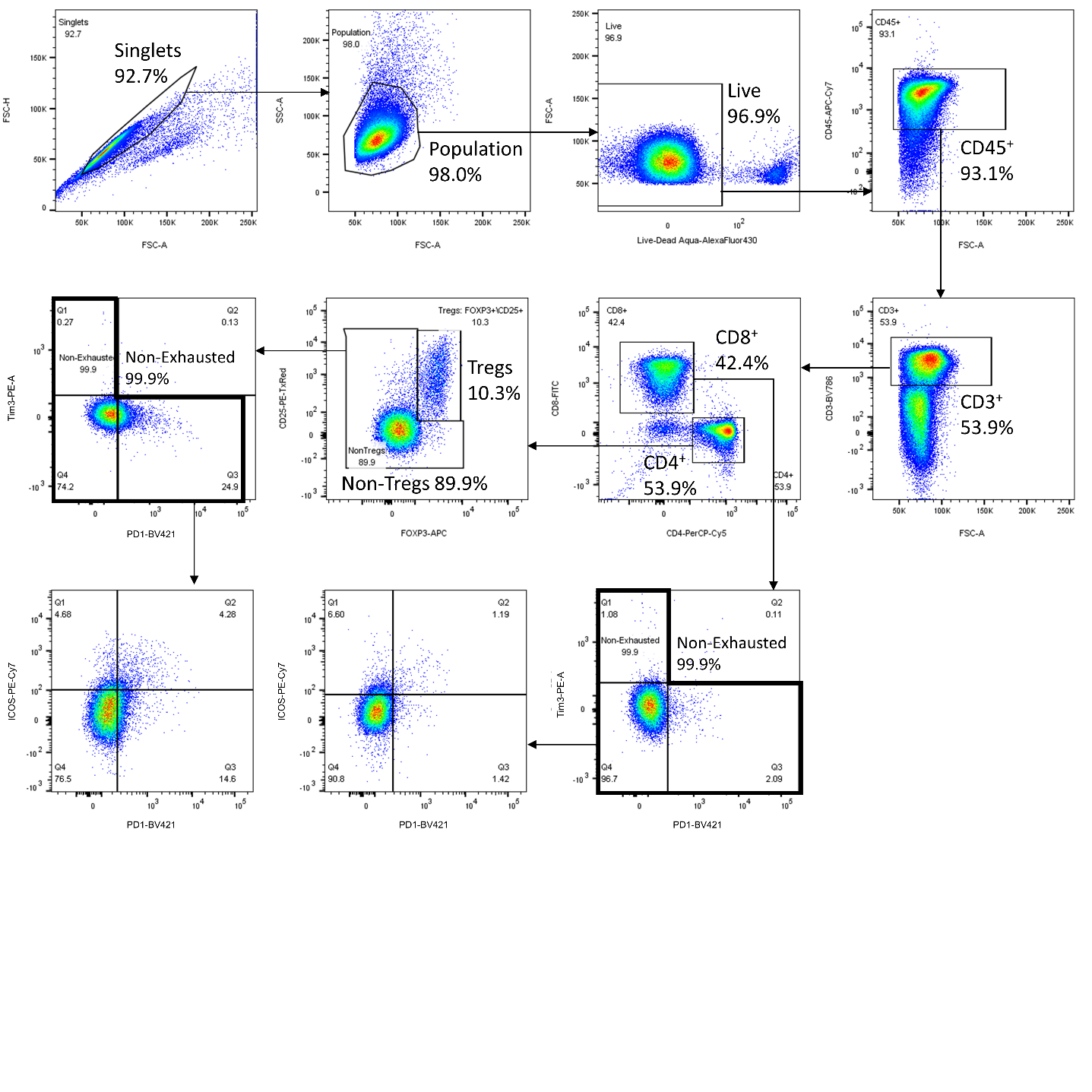


Figure S5. The flow cytometry gating strategy to identify subpopulations of T cells and activation and exhaustion markers.

## NK cell analysis in the tumor and tumor draining lymph node

The population of NK cells in the tumor (**Figure S6a**) and tumor draining lymph node (**Figure S6b**) was evaluated with flow cytometry. NTP treatment did not appear to affect NK cell populations on either day 10 or day 14. Furthermore, overton analysis of IFN-γ with the corresponding isotype also did not show significant differences between the two groups in the tumor or tumor draining lymph node (**Figure S6c**, **d**).


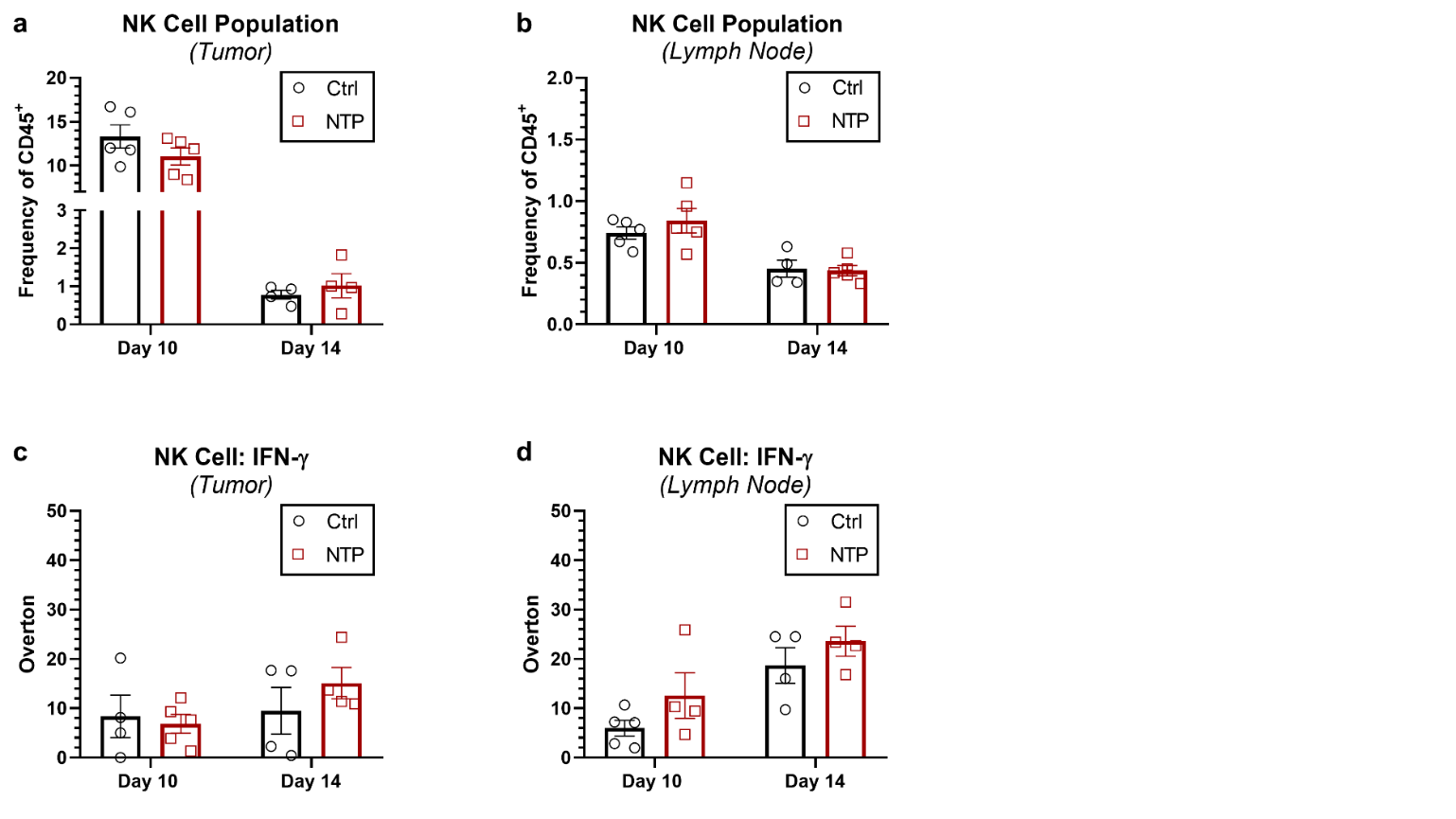


Figure S6. Flow cytometry assessment of NK cells following NTP treatment. The NK cell population in the a) tumor and b) tumor draining lymph node was evaluated on day 10 and 14. Overton analysis of interferon gamma (IFN-γ) expression in the NK cells also did not show significant differences between the NTP-treated and untreated control (Ctrl) groups in the c) tumor or d) lymph node.

5. Detailed Methods

## 5.1 Flow cytometry panels

The following section describes in detail the antibodies and clones used for flow cytometry analysis (**Table S3**). The T cell panel consisted of CD8-FITC (Clone 53-6.7, Biolegend, The Netherlands), Tim3-PE (Clone 5D12, BD Biosciences, Belgium), CD25-PEDazzle549 (Clone PC61, Biolegend), CD4-PerCP/Cy5.5 (Clone GK1.5, Biolegend), ICOS-PE-Cy7 (Clone C398.4A, Biolegend), FOXP3-APC (Clone FJK-16s, Thermofisher Scientific, United States), CD45.2-APC-Cy7 (Clone 104, BD Biosciences), PD-1-BV421 (Clone RMP1-30, Biolegend), LiveDead Aqua (Life technologies, United States), CD3-BV785 (Clone 17A2, Biolegend). The NK cell and DC panel consisted of CD8-FITC (Clone 53-6.7, Biolegend), CD103-PE (Clone 2E7, Thermofisher Scientific), IFN-γ-PE-Dazzle549 (Clone XMG1.2, Biolegend), GranzymeB-PerCP/Cy5.5 (Clone QA16A02, Biolegend), MHC Class II-PE/Cy7 (Clone M5/114.15.2, Biolegend), NK1.1-APC (Clone P136, Biolegend), CD45.2-APC-Cy7 (Clone 104, BD Biosciences), CD11c-BV421 (Clone N418, BD Biosciences), CD11c-BV421 (Clone N418, BD Biosciences), CD3-BV785 (Clone 17A2, Biolegend). The samples obtained from the spleens were stained with the following antibody cocktail, CD8-FITC (Clone 53-6.7, Biolegend), CD103-PE (Clone 2E7, Thermofisher Scientific), CD3-PEDazzle594 (Clone 17A2, Biolegend), CD4-PerCP/Cy5.5 (Clone GK1.5, Biolegend), MHC Class II-PE/Cy7 (Clone M5/114.15.2, Biolegend), FOXP3-APC (Clone FJK-16s, Thermofisher Scientific), CD45.2-APC-Cy7 (Clone 104, BD Biosciences), CD11c-BV421 (Clone N418, BD Biosciences), LiveDead Aqua (Life technologies, United States), CD25-BV785 (Clone PC61, Biolegend).

Table S3. Antibodies used for flow cytometry panels

| **T cell Panel** | | | | |  |
| --- | --- | --- | --- | --- | --- |
| **Fluor** | **Antigen** | **Clone** | **Company** | **Cat No.** | **Dilution** |
| FITC | CD8 | 53-6.7 | Biolegend | 100705 | 1:50 |
| PE | Tim3 | 5D12 | BDBioscience | 566346 | 1:25 |
| PE-TxRd(dzl549) | CD25 | PC61 | Biolegend | 102048 | 1:100 |
| PerCP-Cy5 | CD4 | GK1.5 | Biolegend | 100434 | 1:100 |
| PE-Cy7 | ICOS | C398.4A | Biolegend | 313519 | 1:25 |
| APC | FOXP3 | FJK-16s | Thermofisher | 17-5773-82 | 1:50 |
| APC-Cy7 | CD45 | 104 | BD Bioscience | 560694 | 1:50 |
| BV421 | PD1 | RMP1-30 | Biolegend | 109121 | 1:25 |
| AF430 | L/D Aqua | - | Thermofisher | L34957 | 1:50 |
| BV786 | CD3 | 17A2 | Biolegend | 100231 | 1:100 |
| **DC/NK cell Panel** | | | | |  |
| **Fluor** | **Antigen** | **Clone** | **Company** | **Cat No.** | **Dilution** |
| FITC | CD8 | 53-6.7 | Biolegend | 100705 | 1:50 |
| PE | CD103 | 2E7 | Thermofisher | 12-1031-83 | 1:100 |
| PE-TxRd(dzl549) | IFN-gamma | XMG1.2 | Biolegend | 505845 | 1:100 |
| PerCP-Cy5 | granzyme B | QA16A02 | Biolegend | 372212 | 1:50 |
| PE-Cy7 | MHC-II | M5/114.15.2 | Biolegend | 107630 | 1:50 |
| APC | NK1.1 | PK136 | Biolegend | 108701 | 1:50 |
| APC-Cy7 | CD45 | 104 | BD Bioscience | 560694 | 1:50 |
| BV421 | CD11c | N418 | BD Bioscience | 565452 | 1:50 |
| AF430 | L/D Aqua | - | Thermofisher | L34957 | 1:50 |
| BV786/5 | CD3 | 17A2 | Biolegend | 100231 | 1:100 |
| **Spleen Panel** | | | | |  |
| **Fluor** | **Antigen** | **Clone** | **Company** | **Cat No.** | **Dilution** |
| FITC | CD8 | 53-6.7 | Biolegend | 100705 | 1:50 |
| PE | CD103 | 2E7 | Thermofisher | 12-1031-83 | 1:100 |
| PE-TxRd (dzl549) | CD3 | 17A2 | Biolegend | 100246 | 1:100 |
| PerCP-Cy5 | CD4 | GK1.5 | Biolegend | 100434 | 1:100 |
| PE/Cy7 | MHC-II | M5/114.15.2 | Biolegend | 107630 | 1:50 |
| APC | FOXP3 | FJK-16s | Thermofisher | 17-5773-82 | 1:50 |
| APC-Cy7 | CD45.2 | 104 | BD Bioscience | 560694 | 1:50 |
| BV421 | CD11c | N418 | BD Bioscience | 565452 | 1:50 |
| AF430 | L/D Aqua | - | Thermofisher | L34957 | 1:50 |
| BV786 | CD25 | PC61 | BD Bioscience | 564023 | 1:50 |

## 5.2 Optimization of immunofluorescence staining

Tumor slides were stained with the isotype controls of CRT, CD47, and PD-L1 to check for non-specific staining and determine the optimal primary and secondary antibody dilutions. CRT was optimized previously in the lab and here we demontrated a low amount of non-specific binding (**Figure S7a**). Since the stock concentration of the monclonal CRT antibody and the rabbit IgG isotype control was different, the dilutions were made to keep the final staining concentration the same. The starting concentration for the isotypes of CD47 and PD-L1 were to the same, and we found that for CD47, a 1:100 and a 1:200 dilution was most optimal for primary and secondary staining, respectively (**Figure S7b**), while a 1:200 dilution for both primary and secondary staining was most optimal for PD-L1 (**Figure S7c**). These concentrations were used for staining of all tumor slides.


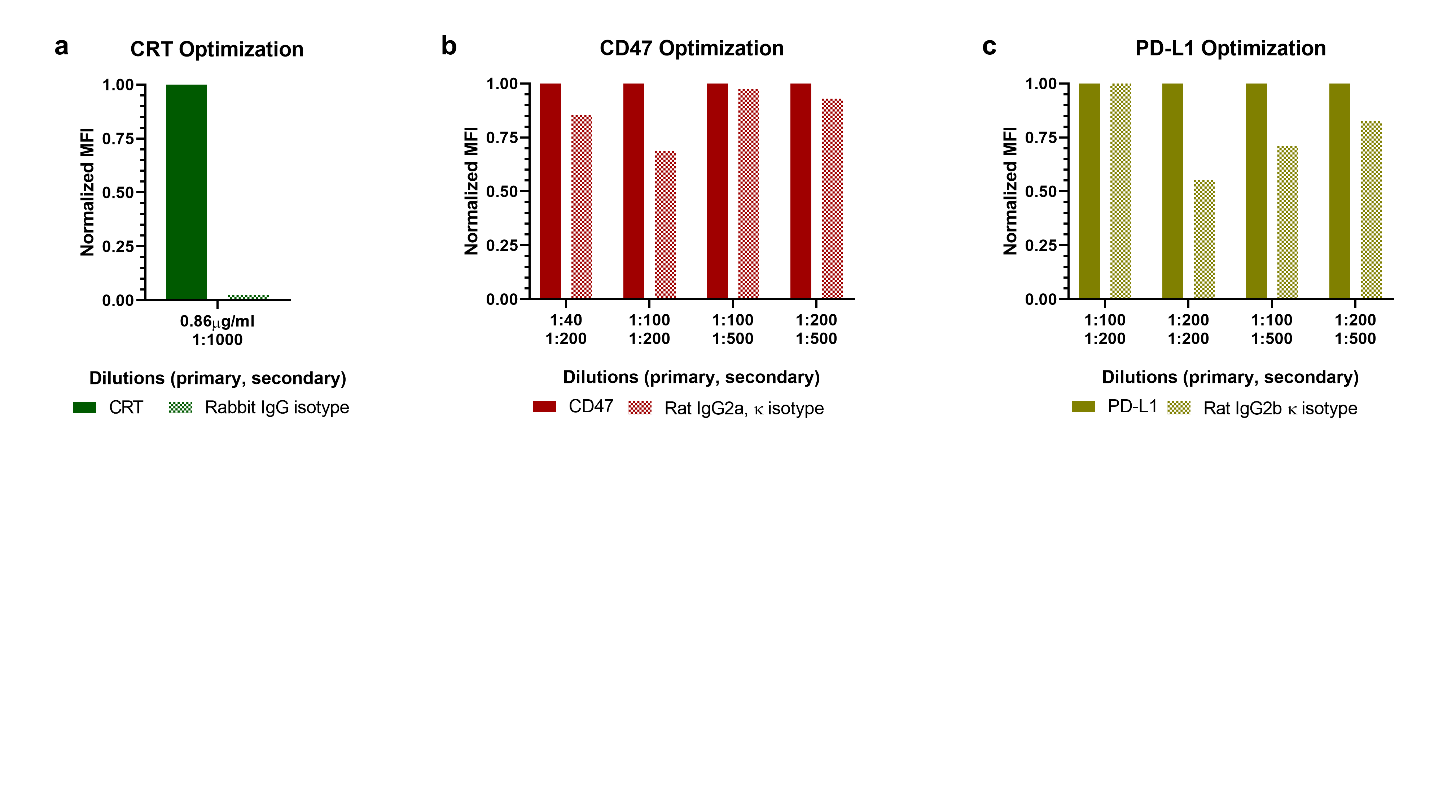


Figure S7. Comparison of primary staining with isotype controls. Data are shown normalized to the primary stain at various dilutions for a) CRT, b) CD47, and c) PD-L1

## 5.3 Computational image processing

Individual nuclei in the images were indexed using connected component analysis and objects smaller than 40 pixels^2^ were removed. The nuclear masks were morphologically dilated using a disk structuring element with diameter of 51 pixels (20.4 µm^2^) to form a cytoplasmic mask. Each pixel of the resulting cytoplasmic mask was indexed to the nearest nuclei. In this way, mean signal intensity and positivity for each cell in the approximated cytoplasm was also measured individually. Signal marker positivity for protein expression were imaged fluorescently using red (TxRED) and green (GFP) channels. To quantify expression of CRT, CD47, and PDL1, the intensity values were corrected for background signal by first saturating the signal to the 95th percentile and subtracting by a one sided low pass filter formed by a Gaussian kernel of 60 pixels in standard deviation. The resultant intensity values for these images were measured using in nuclear and cytoplasmic masks.
